# Abnormally high digestive enzyme activity and gene expression explain the contemporary evolution of a *Diabrotica* biotype able to feed on soybeans

**DOI:** 10.1002/ece3.331

**Published:** 2012-07-19

**Authors:** Matías J Curzi, Jorge A Zavala, Joseph L Spencer, Manfredo J Seufferheld

**Affiliations:** 1 Department of Crop Sciences, University of Illinois at Urbana-ChampaignUrbana, IL, 61801; 2 Cátedra de Bioquímica/INBA (CONICET/UBA), Facultad de Agronomía, Universidad de Buenos Aires, Universidad Católica ArgentinaBuenos Aires, C1417DSE, Argentina; 3 Illinois Natural History Survey, University of Illinois at Urbana-ChampaignChampaign, IL, 61820

**Keywords:** contemporary evolution, landscape heterogeneity, plant defenses, plant–insect interactions, protease inhibitors

## Abstract

Western corn rootworm (*Diabrotica virgifera*) (WCR) depends on the continuous availability of corn. Broad adoption of annual crop rotation between corn (*Zea mays*) and nonhost soybean (*Glycine max*) exploited WCR biology to provide excellent WCR control, but this practice dramatically reduced landscape heterogeneity in East-central Illinois and imposed intense selection pressure. This selection resulted in behavioral changes and “rotation-resistant” (RR) WCR adults. Although soybeans are well defended against Coleopteran insects by cysteine protease inhibitors, RR-WCR feed on soybean foliage and remain long enough to deposit eggs that will hatch the following spring and larvae will feed on roots of planted corn. Other than documenting changes in insect mobility and egg laying behavior, 15 years of research have failed to identify any diagnostic differences between wild-type (WT)- and RR-WCR or a mechanism that allows for prolonged RR-WCR feeding and survival in soybean fields. We documented differences in behavior, physiology, digestive protease activity (threefold to fourfold increases), and protease gene expression in the gut of RR-WCR adults. Our data suggest that higher constitutive activity levels of cathepsin L are part of the mechanism that enables populations of WCR to circumvent soybean defenses, and thus, crop rotation. These new insights into the mechanism of WCR tolerance of soybean herbivory transcend the issue of RR-WCR diagnostics and management to link changes in insect gut proteolytic activity and behavior with landscape heterogeneity. The RR-WCR illustrates how agro-ecological factors can affect the evolution of insects in human-altered ecosystems.

## Introduction

Corn (*Zea mays* L.) is the most important agricultural crop in the United States (US) (USDA-NASS 2011). Six states – Iowa, Illinois, Nebraska, Minnesota, Indiana, and South Dakota – produce nearly 70% of U.S. corn. That production is threatened by both larval and adult activity of the western corn rootworm (WCR), *Diabrotica virgifera virgifera* LeConte (Coleoptera: Chrysomelidae). The WCR is a serious pest of corn across the United States, Canada, and Europe (Gray et al. 2009). WCR larvae can feed and survive on corn roots and a limited number of other grasses (Oyediran et al. 2004; Wilson and Hibbard 2004; Spencer and Raghu 2009). Historically, females expressed high feeding and ovipositional fidelity to cornfields, which – combined with strict larval host requirements and limited motility – made the WCR an ideal candidate for control via annual crop rotation, alternating host corn with the nonhost soybean (*Glycine max*) (Spencer et al. 2009). As a nonhost, soybean fields were not attractive to WCR for feeding or oviposition. When WCR eggs are laid in a cornfield destined for rotation to soybean the following year, the larvae that emerge from those eggs starve among nonhost soybean roots. Until the mid-1990s, annual crop rotation between corn and soybean was an effective pest management strategy for reducing WCR damage (Levine et al. 2002). Crop rotation virtually eliminated the need for corn rootworm-targeted chemical control and it was widely adopted. However, reliance on a simple, two-crop rotation also decreased landscape heterogeneity in East-central Illinois. This practice significantly increased the probability that any WCR adults leaving a cornfield would encounter soybean. Because *ca*. 90% of cornfields were annually rotated to soybean and 98% of soybean fields were rotated to corn, laying eggs in soybean fields nearly assured that WCR eggs would later hatch in a cornfield. (Levine and Oloumi-Sadeghi 1996; Isard et al. 2000; Levine et al. 2002; Mabry and Spencer 2003; Schroeder et al. 2005; Spencer et al. 2005). This circumstance selected for high motility (Mabry et al. 2004; Knolhoff et al. 2006, 2010a), a trait thought to be the enabling adaptation of rotation-resistant WCR populations (RR WCR). Individuals from the highly mobile RR WCR populations are more likely to enter, reside in, feed, and oviposit in soybean fields (reviewed in Spencer et al. 2009). Consequently, larvae emerging from eggs originally laid in soybean fields can damage the rotated corn planted in those fields the following year.

Although crop rotation remains effective in many regions, the range of the RR WCR (currently found in the states of Illinois, Indiana, and portions of Ohio, Wisconsin, Michigan, Iowa, and Ontario Province in Canada) continues to expand across the US Corn Belt (Gray et al. 2009). Evidence of WCR injury in rotated corn or the presence of abundant WCR adults in soybean fields is associated with the likely presence of RR WCR populations and is used to improve management (O'Neal et al. 1999, 2001). Attempts to identify diagnostic characteristics that could help detect individual RR WCR have made progress, but remain impractical (Knolhoff et al. 2006, 2010a,b; Garabagi et al. 2008). Methods to characterize RR WCR would advance the study of their evolution and improve grower management options.

RR WCR adults cannot survive solely on a diet of soybean tissue (Mabry and Spencer 2003) but can tolerate a diet that alternates between corn and soybean tissues (Mabry et al. 2004). In the field, RR WCR periodically move back and forth between cornfields and soybean fields (Isard et al. 2000). Although we have no direct evidence of WCR residence in soybean beyond *ca*. 24 h, residence times of up to 4 days seem plausible based on survival studies by Mabry and Spencer (2003) and Mabry et al. (2004). RR WCR adults must either avoid or tolerate soybean plant defenses to feed and temporarily colonize soybean fields. Soybeans are particularly well defended against Coleopteran insects by cysteine protease inhibitors (CystPIs) that are regulated by at least two constitutive and two wound-inducible genes (Botella et al. 1996; Misaka et al. 1996; Zhao et al. 1996). Inducible soybean CystPI targets cathepsin L endopeptidases, the main digestive proteases in the WCR midgut, inhibiting more than 90% of the proteolytic activity (Koiwa et al. 2000). Protease inhibitors impair the ability of insects to digest proteins and assimilate amino acids required for their growth, development, and reproduction (Birk 2003; Zavala et al. 2004). Soybean herbivory induces CystPI activity in foliage, decreasing gut cysteine protease activity in WCR adults and forcing them to feed on foliage with low CystPI activity (Zavala et al. 2008). Soybean CystPI activity reduces growth and survival of both WCR larvae and adults (Zhao et al. 1996; Koiwa et al. 2000; Kim and Mullin 2003; Lalitha et al. 2005).

Broadly, Coleopterans respond to high dietary CystPI activity by changing their feeding site on the plant or via biochemical changes in the gut (Zhu-Salzman et al. 2003; Zavala et al. 2009). Colorado potato beetle (*Leptinotarsa decemlineata*) and the cowpea bruchid (*Callosobruchus maculatus*) compensate for cysteine protease inhibition by increasing the expression and activity of gut digestive proteases (Bolter 1995; Cloutier et al. 2000; Zhu-Salzman et al. 2003; Gruden et al. 2004; Moon et al. 2004). A diet containing soybean CystPI strongly induced cathepsin B-like protease transcripts in cowpea bruchids (Koo et al. 2008). Protease activity in the WCR gut is regulated by at least five cathepsin L-like and two cathepsin B-like protease genes, which are susceptible to inhibition by soybean CystPIs (Koiwa et al. 2000; Bown et al. 2004; Siegfried et al. 2005). We predict that compared with rotation-susceptible wild-type (WT) adults that rarely enter soybean fields, RR WCR should have constitutively high gut cysteine protease activity or the ability to significantly increase activity while feeding on soybean foliage. In the last 15 years, previous work to document differences between the WT and RR phenotypes of WCR has described only behavioral differences related to greater motility (Levine and Oloumi-Sadeghi 1996; Levine et al. 2002; Mabry and Spencer 2003; Spencer et al. 2005; Knolhoff et al. 2006, 2010a). Garabagi et al. (2008) tested whether expression of the cyclic GMP-dependent protein kinase gene (a homolog of the *foraging* gene implicated in movement and foraging in *Drosophila* and honeybees) was associated with RR WCR behavior; however, the differences in kinase gene expression showed no direct link between RR and behavior of lab-reared insects. Further attempts to identify eco-physiological, biochemical, and molecular differences between phenotypes and to explain how RR WCR can feed on soybean foliage have proved elusive (Miller et al. 2006, 2007; Knolhoff et al. 2010b).

To assess the role of digestive cysteine proteases in the adaptation of RR WCR adults to soybean herbivory, field populations from locations that harbor predominately either the WT or RR WCR biotype were collected from cornfields in Illinois, Iowa, Missouri, and Nebraska. We conducted field and laboratory experiments using WT and RR WCR adults that fed on either corn silks or soybean foliage to investigate the apparent RR WCR tolerance of soybean CystPI. Specifically, we examined (1) whether RR WCR adults survived longer and fed more on soybean foliage than WT WCR beetles and (2) whether there were differences in cysteine protease activity and gene expression in the guts of RR WCR versus WT WCR adults that fed on either corn silks or soybean foliage. Our data indicate that altered protease activity and expression likely allow RR WCR adults to tolerate soybean CystPIs, an adaptation that would facilitate prolonged periods of feeding and oviposition in soybean fields.

## Materials and methods

### Sample collection

Field and laboratory experiments were performed with WT and RR WCR adults collected from foliage and silks/ears in cornfields using funnel-topped jars or a DC Insect Vacuum (BioQuip Products, Rancho Dominguez, CA 90220, USA). RR WCR populations were collected at three locations: Urbana (Champaign County), IL (40°9′14″N, 88°8′40″W); LaSalle (LaSalle county), IL (41°21′23″N, 89°4′1″W); and Minonk (Woodford County), IL (40°51′26″N, 89°0′26″W) (Fig. S1). In addition, three WT WCR populations were collected at Concord (Dixon County), NE (42°23′39″N, 96°57′23″W); Ames (Story County), IA (42°3′8″N, 93°32′6″W); and Higginsville (Lafayette County), MO (39°07′09″N, 93°49′42″W) (Fig. S1). After field collection and during transit to the laboratory, each population was maintained on corn tissues (silks and immature ears) from their field of origin. In all the locations, the composition of the collected insects was 60–70% females. Populations were maintained separately in 30 × 30 × 30 cm wire-screen cages. Once in the laboratory, cages were held in growth chambers at 24°C, 70–90% RH, and 14:10 h (L:D) photoperiod. All populations were fed with the same diet consisting of corn silks and immature sweet corn kernels (*Zea mays*, variety Sugar Buns) grown in an experimental field at the University of Illinois in Urbana, IL. The total interval between field collection and the beginning of experiments, which included preexperiment diet standardization on sweet corn, was 5–7 days.

### Field experiments

To determine WCR survivorship on soybean after herbivory under field conditions, soybeans (*Glycine max* cultivar 93B15, Pioneer Hi-Bred, Des Moines, IA, USA) were grown at the University of Illinois at Urbana-Champaign. Nine undamaged vegetative stage soybean plants were selected; each was infested with 10 WCR adults from either Urbana (IL), LaSalle (IL), or Ames (IA). This experiment was performed twice with a total of 18 plants and 180 beetles (10 beetles per plant). Beetles were fed corn silks for 5 days before they were placed on a soybean plant enclosed with a fine mesh bag in the field. The number of living and dead beetles was recorded at 1, 1.5, 3, and 4 days after infestation. Survivorship was analyzed using the Kaplan–Meier method to construct a survival distribution curve for each population (Lee and Wang 2003). Then, the Logrank test was used to compare survival distributions between populations at α = 0.05 (SAS LIFETEST procedure, SAS Institute Inc., 2009).

To determine cysteine protease activity in WCR under field conditions, a second independent experiment was conducted. Soybean plants were infested with beetles from the three field-collected populations as explained above. Nine soybean plants were selected; each was infested with 10 WCR adults from either Urbana (IL), LaSalle (IL), or Ames (IA). *This experiment was performed twice (a total of 18 plants and 180 beetles, 10 beetles per plant)*. Beetles were collected at 0 (pretreatment control, fed on corn silks), 8, 24, 36, and 72 h after soybean infestation and dissected to determine midgut cysteine protease activity. After infestation, undamaged and damaged leaves were harvested daily from soybean plants for cysteine protease inhibitor (CystPI) activity analysis. The complete procedure is detailed below in a separate section, as it was the same procedure used for the laboratory experiments.

Finally, a third experiment was conducted to determine CystPI activity induced by WCR herbivory in soybean plants growing in the field. Documenting CystPI induction following WCR herbivory was required to show that WCR gut protease activity was related to soybean plant defense. Four plants were infested with 5 RR WCR adults (Urbana population) each. Bagged beetles were allowed to feed for 0, 1, 2, 3, and 4 days at the same leaf position; control plants were maintained identically but were not infested with WCR. Leaves from undamaged control and infested plants were collected at each time point of WCR herbivory (four plants per time point, for a total of 20 plants). Leaf samples were flash frozen in liquid nitrogen and ground to a fine powder; the unit of replication for statistical analysis was the individual plant (*n* = 4). Activity of cysteine protease inhibitors (CystPI) in either corn-silk or soybean-leaf powder was measured against papain by following the release of p-nitroaniline (pNA; 37°C for up to 20 min at 405 nm) after adding the synthetic substrate p-Glu-Phe-Leu-pNA (Zavala et al. 2008). Data analysis was performed by analysis of variance (ANOVA) using a completely randomized design, followed by Fisher's protected LSD post hoc comparisons (α = 0.05).

### Laboratory experiments

Three WT (Ames, IA, Higginsville, MO, and Concord, NE) and two RR (Urbana and Minonk, IL) WCR populations collected from cornfields and maintained on corn-silk diet were used in three independent laboratory experiments with different objectives: (1) measurement of WCR survivorship on a soybean diet; (2) evaluation of soybean damage due to WCR feeding; and (3) determination of digestive cathepsin protease activity and gene expression analysis. Soybean (cultivar Williams 82) was grown in 30-cm-diameter pots, in a greenhouse facility (light intensity of 1200–1500 μmol m^−2^ s^−1^) at the University of Illinois at Urbana-Champaign. Each pot was enclosed in a mesh bag and infested with 30 beetles of mixed sex (the sex ratio observed in the field was used). To ensure soybean consumption on the first day of treatment, food was removed from the cages containing WCR populations for 48 h preceding the start of soybean herbivory treatments, leaving only the water supply. A starvation period was important for the WT populations, as they avoid eating soybean unless they are food deprived for a considerable period of time. The procedure described above was identical for all laboratory experiments.

### Soybean feeding and survivorship tests

Soybean damage tests were conducted using four replicates (pots) of vegetative stage soybean plants (4–5 weeks old) per population. Each pot was enclosed in an insect-proof mesh bag and infested with 30 beetles as explained above. Pots were kept in a growth chamber at 24°C, 70–90% RH on a 14:10 (L:D) photoperiod. After 7 days, leaves were removed from each plant and insect damage was measured taking into account the number of leaflets damaged and the area of tissue eaten from each leaflet. A visual estimate of the percentage damage (missing leaf tissue) was used to assign leaflets to one of five damage classes. To designate the damage classes, the percentage of missing tissue was subdivided into five ranges (very low, <10% of missing tissue; low, 10–25%; medium, 26–40%; high, 41–70%; very high, >70%). The value halfway between the lower and upper boundaries of a given class was used as the damage coefficient for that class. The level of damage within each experimental unit (pot) was calculated as the sum of the number of leaflets grouped in each class, multiplied by the coefficient for that class. To account for differential survival rates, the number of live beetles in each pot was recorded daily, and the damage value of a given pot was divided by the mean survival time of beetles in that pot. The calculated value was called the soybean damage index (SDI). Data analysis was performed using SAS software, version 9.2 (SAS Institute Inc, 2009). Soybean damage was analyzed by ANOVA using a completely randomized design and the GLM procedure, followed by Fisher's protected LSD post hoc comparisons (α = 0.05).

To determine the survivorship of soybean-feeding WCR, 60 adults from each population were used to infest three soybean pots; the number of dead beetles was recorded daily for 4 days. Survivorship was analyzed using the Kaplan–Meier method to construct a survival distribution curve for each population (Lee and Wang 2003). Then, the Logrank test was used to compare survival distributions between populations at α = 0.05 (SAS LIFETEST procedure, SAS Institute Inc., 2009).

### Cysteine protease activity in adult WCR guts

WCR beetles from all populations used in both field and lab experiments were sampled at 0 (pretreatment control, fed on corn silks), 8, 24, 36, and 72 h after soybean infestation and dissected to determine midgut cysteine protease activity. For each WCR population at each time point, midguts were removed from 10 beetles and combined to create one independent replicate. We used a total of 30 beetles to create three independent replicates of 10 guts each. Gut tissue was stored at −80°C. The composite beetle gut samples were pulverized in liquid nitrogen with a mortar and pestle. Protease extraction and activity assays were conducted according to Zavala et al. (2008), with minor modifications. WCR midgut proteases were extracted by homogenization of 1.5 μL/mg gut tissue with 30-mM Tri-K citrate (pH 6.0) and incubated on ice for 30 min. The suspension was centrifuged at 12,000 g for 15 min at 4°C, and the resulting supernatant used to determine WCR gut protease activity. The chromogenic substrate p-Glu-Phe-Leu-pNA was used to quantify cathepsin L protease activity (Filippova et al. 1984). This substrate is specific for cathepsin L proteases, although some proportion can be hydrolyzed by cathepsin B proteases. To avoid nonspecific cathepsin B activity, samples were preincubated with CA-074, a strong and highly specific cathepsin B inhibitor (Murata et al. 1991). The reaction buffer [0.1 M NaH_2_PO_4_, 0.3 M KCl, 0.1 mM EDTA, and 3 mM dithiothreitol (pH 6.0)] and 5 μL of the enzyme extract were added to the wells on a microtiter plate. The inhibitor CA-074 was added to a final concentration of 1 μM, and the mixture was incubated for 10 min at 37°C. Subsequently, the enzymatic reaction was started with the addition of p-Glu-Phe-Leu-pNA diluted in 30% DMSO and 70% reaction buffer to a final concentration of 76 μM of substrate in the reaction mixture. The hydrolysis of p-Glu-Phe-Leu-pNA releases p-nitroaniline (pNA), which can be measured with a spectrophotometer at 405 nm (Filippova et al. 1984). A calibration curve was constructed using increasing concentrations of pNA to convert from OD units to nanomoles of pNA (R^2^ = 0.999). Absorbance at 405 nm from wells on the microtiter plate containing gut extracts and reaction mixture was measured at 20-s intervals for 30 min at 37°C. Initial rates of hydrolysis were estimated from the slopes of the resulting absorbance versus time graphs. Protease activity was calculated as nanomoles of pNA released per mg of fresh gut tissue per minutes. Cathepsin L protease activity was analyzed by ANOVA using a 5 × 5 (population × time) factorial design, followed by Fisher's protected LSD post hoc comparisons.

Cathepsin B and L cysteine proteases are responsible for digestive proteolysis in WCR (Bown et al. 2004; Kaiser-Alexnat 2009). Substrate specificity in cathepsin L endopeptidase is normally determined by the amino acid residue at the P2 position, and this protease preferentially cleaves peptides bonds involving hydrophobic residues find in the substrate p-Glu-Phe-Leu-pNA (Barrett et al. 1998). However, cathepsin B protease can also degrade the substrate p-Glu-Phe-Leu-pNA at a low rate. To differentiate cathepsin L activity from cathepsin B activity, we used the specific inhibitor of cathepsin B, CA-074 (L-3-trans-(Propylcarbamyl) oxirane-2-carbonyl)-L-isoleucyl-L-proline (Murata et al. 1991). As CA-074 is not specific for cathepsin B at concentrations >10^−6^M, with IC50 values estimated as 2.2 × 10^−9^M and 1.7 × 10^−4^M for rat cathepsin B and cathepsin L, respectively (Murata et al. 1991), we assayed 10^−6^M of CA-074 with the chromogenic substrate p-Glu-Phe-Leu-pNA (Bown et al. 2004). Although we did not use a specific substrate for cathepsin B, it is possible to consider the activity levels inhibited by CA-074 as an approximation of the relative activity of cathepsin B activity across samples. The inhibition effect of CA-074 for each population and time point was analyzed using a paired t-test, to test whether the effect of the inhibitor was different from zero.

To ensure that cysteine protease activity and expression were analyzed under identical conditions, the beetles for these two analyses were collected simultaneously from the same pots (see below).

### Gene expression analysis

Digestive cathepsin gene expression analysis was conducted in gut tissue of WT and RR WCR beetles for laboratory experiments. To determine the expression of genes related to cathepsins L and B, five cDNA sequences encoding L-like WCR cathepsins, DvRS5, DvRS29, DvCAL1, DvRS30, and RvRS33, and two sequences encoding B-like WCR cathepsins, DvRS40 and DvRS6 (Koiwa et al. 2000; Bown et al. 2004), were analyzed in this study. Beetles were collected at 0 (pretreatment control, fed on corn silks), 8, and 24 h after soybean infestation and dissected. Guts were flash frozen in liquid nitrogen and stored at −80°C with the addition of 50 μL RNAlater (Ambion). For each population and treatment, we used a total of 15 beetles to create three independent replicates of five guts each. Total RNA was extracted from frozen gut tissue.

The isolation of total RNA was conducted using the E.Z.N.A. Total RNA Kit I (Omega Bio-Tek) according to the manufacturer's instructions, including the DNase on-column treatment. For each sample, 2 μg of RNA were converted to cDNA by using the SuperScript III first-strand synthesis system for RT-PCR according to the manufacturer's instructions (Invitrogen). A dilution 1:50 of the reverse transcription product was used as a template to amplify cathepsin cDNA fragments with the appropriate primer combinations. PCR reactions were conducted using a Taq DNA polymerase with ThermoPol buffer, according to the manufacturer's instructions (New England Biolabs). PCR reactions were standardized and optimized independently for each sequence and pair of primers. A series of calibration runs using the same concentration of cDNA template were performed to determine the number of PCR cycles at which each sample reached saturation. Subsequently, we determined the number of cycles that gave products that were still in the exponential range of amplification before the reaction plateau. Briefly, after determining the numbers of cycles to reach saturation, a series of PCR reactions with decreasing numbers of cycles were done and their products were evaluated by band intensity until all bands obtained were unsaturated. To further confirm that the number of cycles chosen was correct, a PCR was conducted with the number of cycles reduced by one to check whether the bands were more diffuse. This procedure assured that the number of cycles chosen as optimal was not in the final lag portion but in the exponential section of the amplification curve. Calibrated PCR reactions consisted of a denaturizing step at 94°C for 2 min, followed by annealing at 50°C for 45 sec, and extension at 68°C for 1 min. The optimal number of cycles for fragments DvRS5, DvRS29, DvRS40, DvCAL1, DvRS6, DvRS30, and DvRS33 was 21, 27, 27, 30, 30, 35, and 35, respectively. The same protocol was applied to elongation factor EF-1a that served as the internal standard gene to determine equal amplification of cDNA (Knolhoff et al. 2010b). The optimal number of PCR cycles for EF-1a was 30. For each sample, the target gene and internal standard gene (EF-1a) were separately amplified. Products were then loaded on a gel to confirm single PCR products and for analysis of band intensity. The amplified cDNA fragments were purified from agarose gels by using a QIAquick Gel Extraction Kit (Qiagen) and sequenced. The gene names, accession numbers, and primer sequences are provided in the supporting information (Table S1). Sequences of the amplified fragments used for expression analysis are provided in Table S2.

Band intensity was determined with image analysis software (Image Lab, Bio-Rad). Prior to statistical analysis, the band intensity values generated by image analysis software for each gene were normalized to the intensity of elongation factor EF-1a obtained for each individual cDNA sample, to correct for variability in purification of RNA, synthesis of cDNA and PCR runs. Band intensity values were analyzed by ANOVA using a 5 × 3 (population x time) factorial design with random blocks, followed by Fisher's protected LSD post hoc comparisons. Each block consisted of an individual gel loaded with a complete replication of populations and treatment combinations for each gene (5 populations, 3 time points, total = 15 samples) plus EF-1a to account for variability between gels. There were three blocks for each gene in the statistical design (as there were three independent replications for each population and time combination). Data analysis was performed using the MIXED procedure included in SAS software, version 9.2 (SAS Institute Inc.). The *P*-values of all pairwise comparisons between the main effect of WCR populations and for each individual time point are provided in Table S3.

## Results

**Figure 1:**
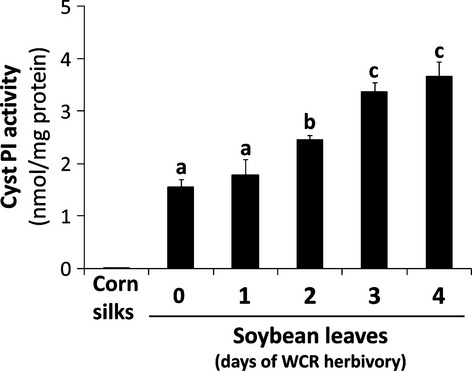
CystPI activity in fully expanded soybean leaves and corn silks grown under field conditions. CystPI activity (mean ± SE) of soybean leaves was determined before (0 days) and after 1, 2, 3, and 4 days of WCR feeding on foliage. Bars bearing the same letter are not significantly different at *P* < 0.05.

To determine the defensive response of soybeans to adult WCR herbivory, we measured constitutive and inducible levels of CystPI in damaged leaves. We also measured CystPI activity in corn silks, a common food for all WCR adults, but found no CystPI activity in corn silk extracts. WCR damage increased CystPI activity in field soybean foliage from 1.55 nmol/mg in undamaged leaves to 3.66 nmol/mg after 4 days of herbivory (*P* < 0.01; Fig. 1).

**Figure 2:**
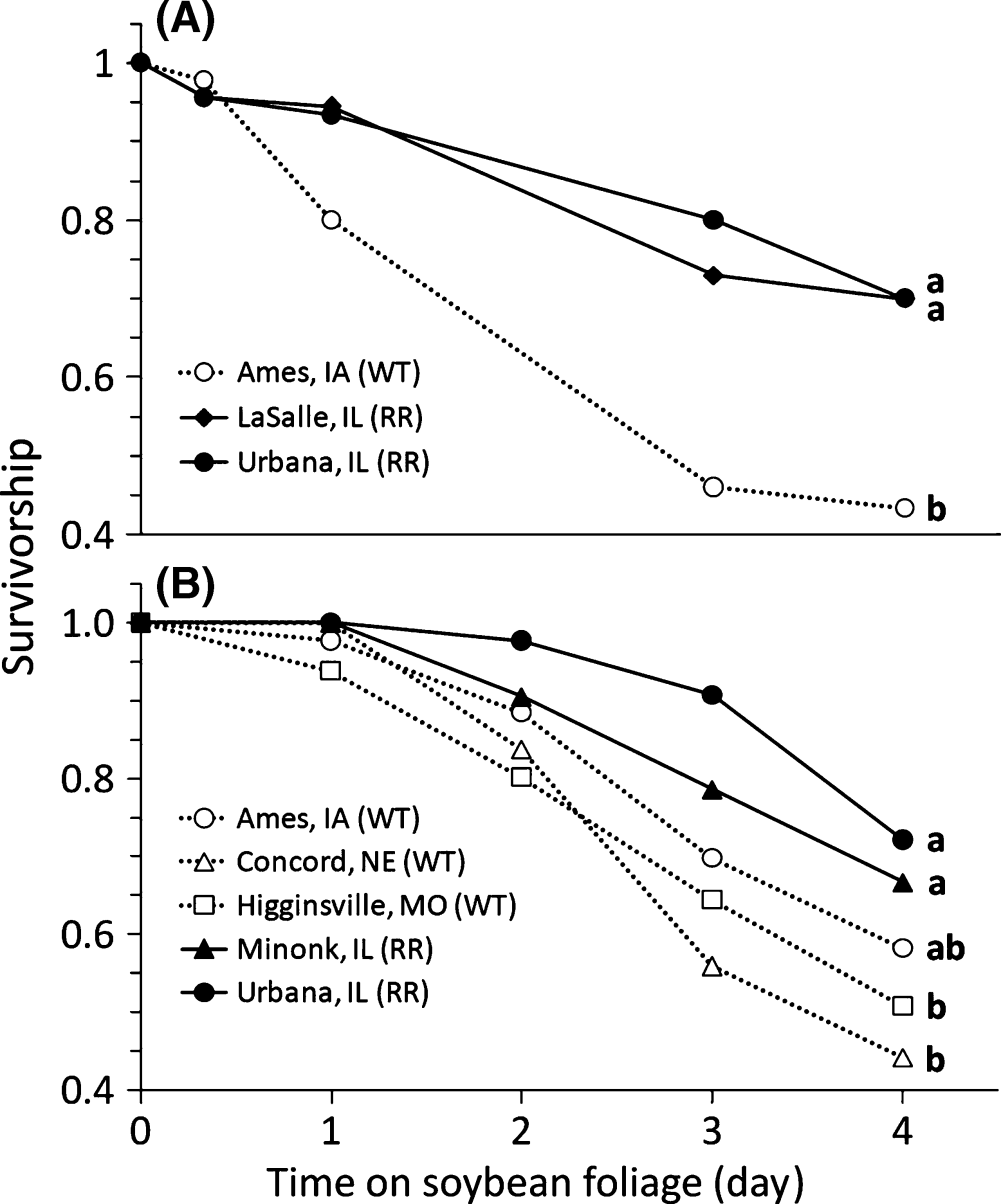
Survival of WCR adults feeding on soybeans. (A) Experiments were conducted with soybeans grown in the field. (B) Experiments conducted under laboratory conditions. Dotted lines with open markers represent wild-type (WT) populations and filled markers with solid lines indicate rotation-resistant (RR) populations. For field experiments, soybean plants were infested with 90 beetles from each population. For laboratory experiments, soybean plants were infested with 60 beetles from each population and maintained at 24°C, 70–90% RH, and 14:10 h (L:D) photoperiod. A survival distribution curve for each population was constructed using the Kaplan–Meier method, and the differences between survival functions were analyzed using the Logrank test. Time 0 corresponds to pretreatment beetles fed on corn silks. Curves bearing the same letter are not significantly different at *P* < 0.05.

Although dietary soybean CystPIs increased Coleopteran mortality (Zhao et al. 1996; Koiwa et al. 2000), WCR adults from RR populations readily fed on soybean tissues (Levine et al. 2002). To investigate whether WCR RR populations can tolerate soybean herbivory, we determined the survivorship of both WT and RR populations feeding on soybean foliage during a period of 4 days. Our field experiments demonstrated that both RR populations (Urbana and LaSalle, IL) survived longer on soybean foliage than the WT (Iowa) population (*P* < 0.01; Fig. 2A). Laboratory experiments yielded similar results; adults of the RR populations displayed higher survivorship than two of the WT populations (Nebraska and Missouri; *P* < 0.01 and *P* < 0.05 compared with Urbana and Minonk, respectively). Adults of the Iowa population (WT) exhibited intermediate survivorship levels that were not different from any of the other populations (*P* > 0.05; Fig. 2B).

**Figure 3:**
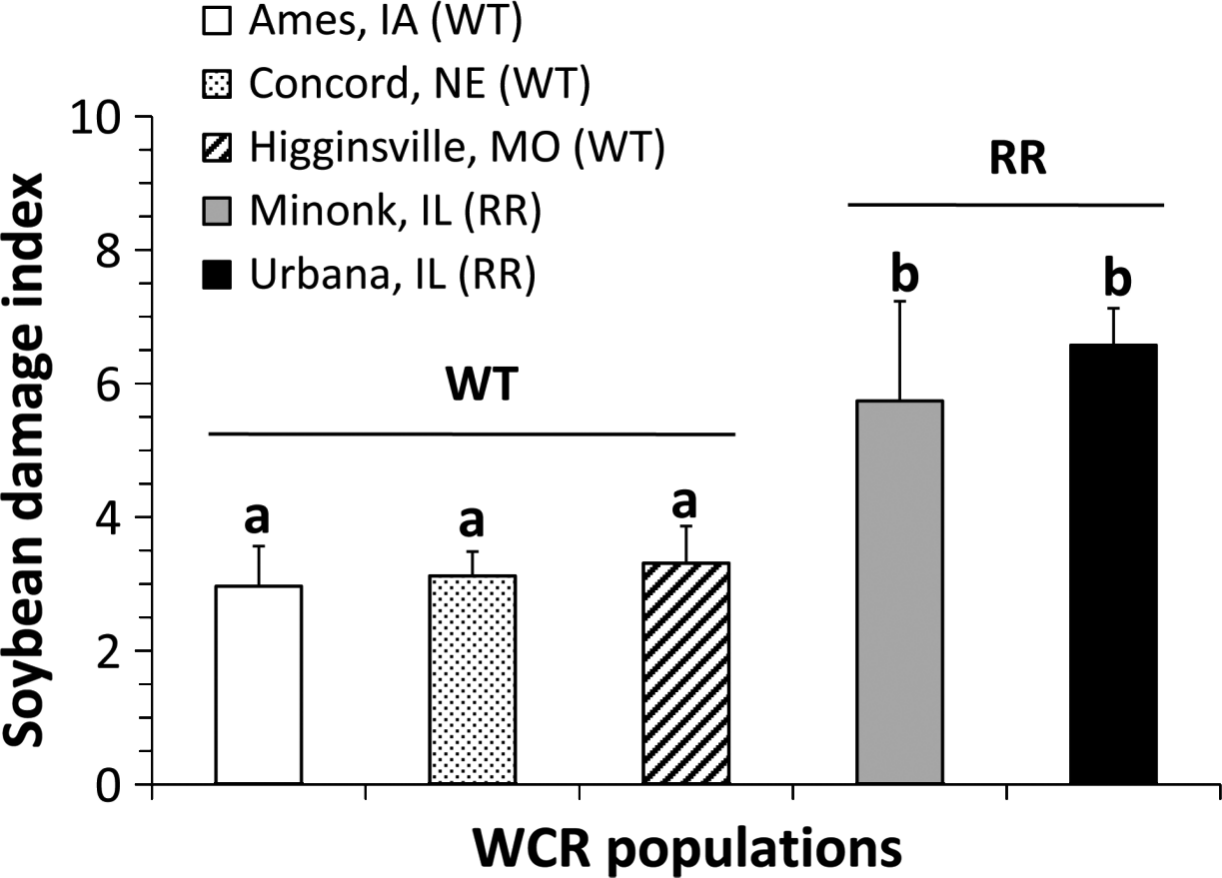
Soybean Damage Index (SDI) for WCR adults. Three wild-type (WT) WCR populations (IA, NE, and MO) and two rotation-resistant (RR) WCR populations (IL) were analyzed. Values for each population represent the average SDI (mean ± SE) of four independent pots, each one containing four vegetative stage soybean plants infested with 30 WCR adults. Pots were maintained at 24°C, 70–90% RH, and 14:10 (L:D) photoperiod. After 7 days of infestation, soybean damage was measured and adjusted by the mean WCR survival time on each pot to obtain SDI values. Bars bearing the same letter are not significantly different at *P* < 0.05.

As beetles from RR populations survived longer on soybean foliage than WT WCR, RR beetles may have greater tolerance of CystPIs and a capacity for prolonged feeding on soybean plants compared with WT WCR. As expected, beetles from RR populations consumed more soybean foliage during 7 days of herbivory than WCR from the WT populations (*P* < 0.05 for all WT populations compared with RR populations; Fig. 3). As the calculation of soybean damage index (SDI) values accounted for the number of living WCR in each pot, the values reflect individual WCR damage. These results suggest that RR beetles better tolerate the effect of soybean CystPIs. We hypothesize that compared with WT, RR WCR may have higher activity levels of cathepsin L cysteine proteases, which are the main enzymes responsible for digestive proteolysis in WCR (Koiwa et al. 2000; Bown et al. 2004).

**Figure 4:**
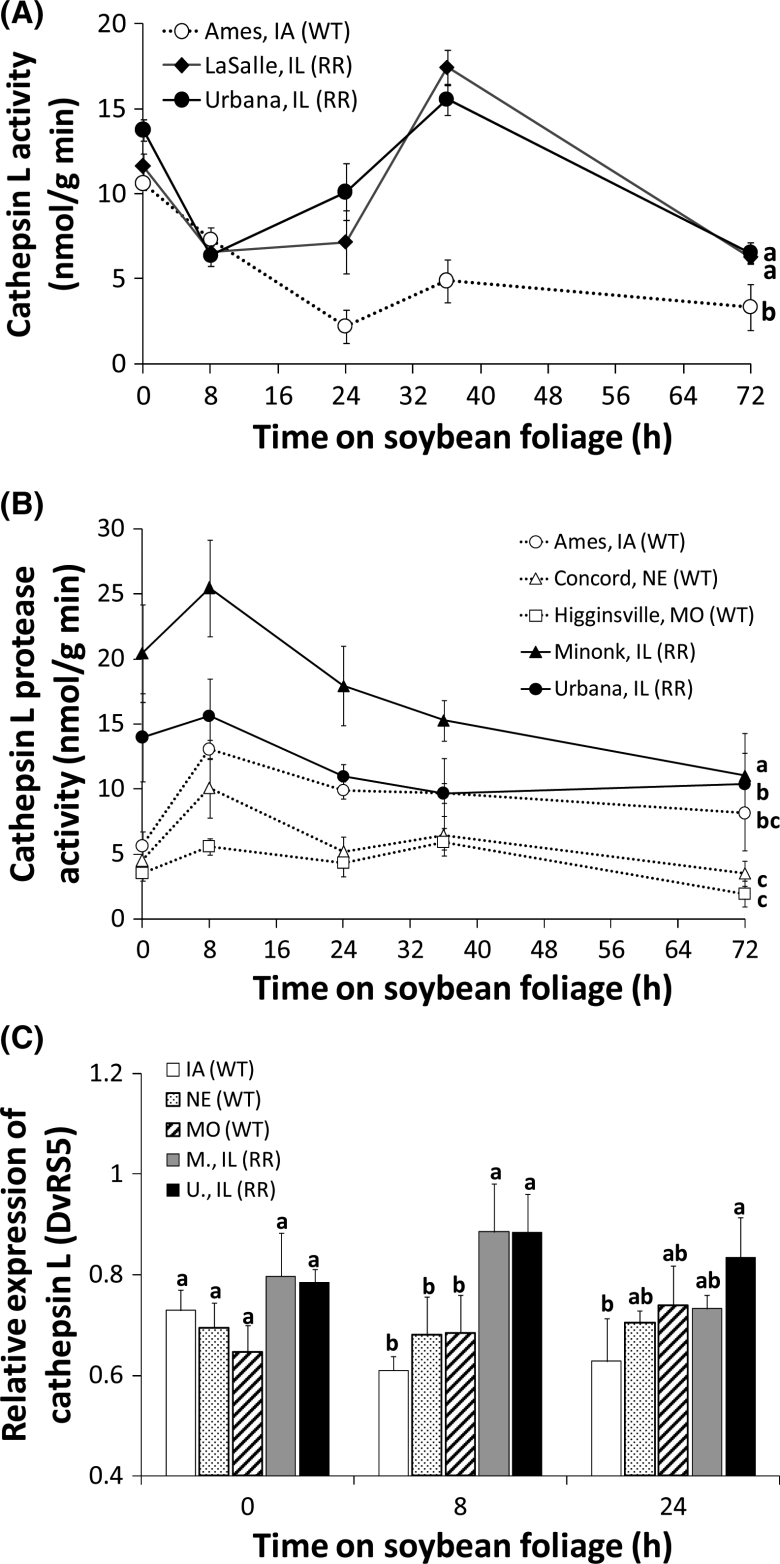
Protease activity and gene expression in guts of RR and WT WCR. (A) Cathepsin L-like protease activity in guts of WCR adults fed on soybeans in field conditions. (B) Cathepsin L-like protease activity in guts of WCR adults fed on soybean foliage in laboratory conditions. Wild-type (WT) populations are represented with open markers and dotted lines, whereas rotation-resistant (RR) populations are represented by filled markers and solid lines. Insects were dissected and gut samples were collected at 8, 24, 36, and 72 h after the initiation of the experiment. Time 0 corresponds to pretreatment beetles fed on corn silks. Markers represent the mean ± SE of three independent samples each containing guts from 10 WCR. Lines bearing the same letter are not significantly different between populations at *P* < 0.05. (C) Quantitative RT-PCR expression analysis of cathepsin L-like (protease gene DvRS40; accession number AJ583508) in guts of WCR adults fed for 0, 8, and 24 h on soybean foliage. Time points for each population represent three replicates, each derived from five beetles. Values represent the band intensity generated by image analysis software for the results of quantitative RT-PCR relative to EF-1a (mean ± SE). Bars bearing the same letter within each time point are not significantly different between populations at *P* < 0.05.

**Figure 5:**
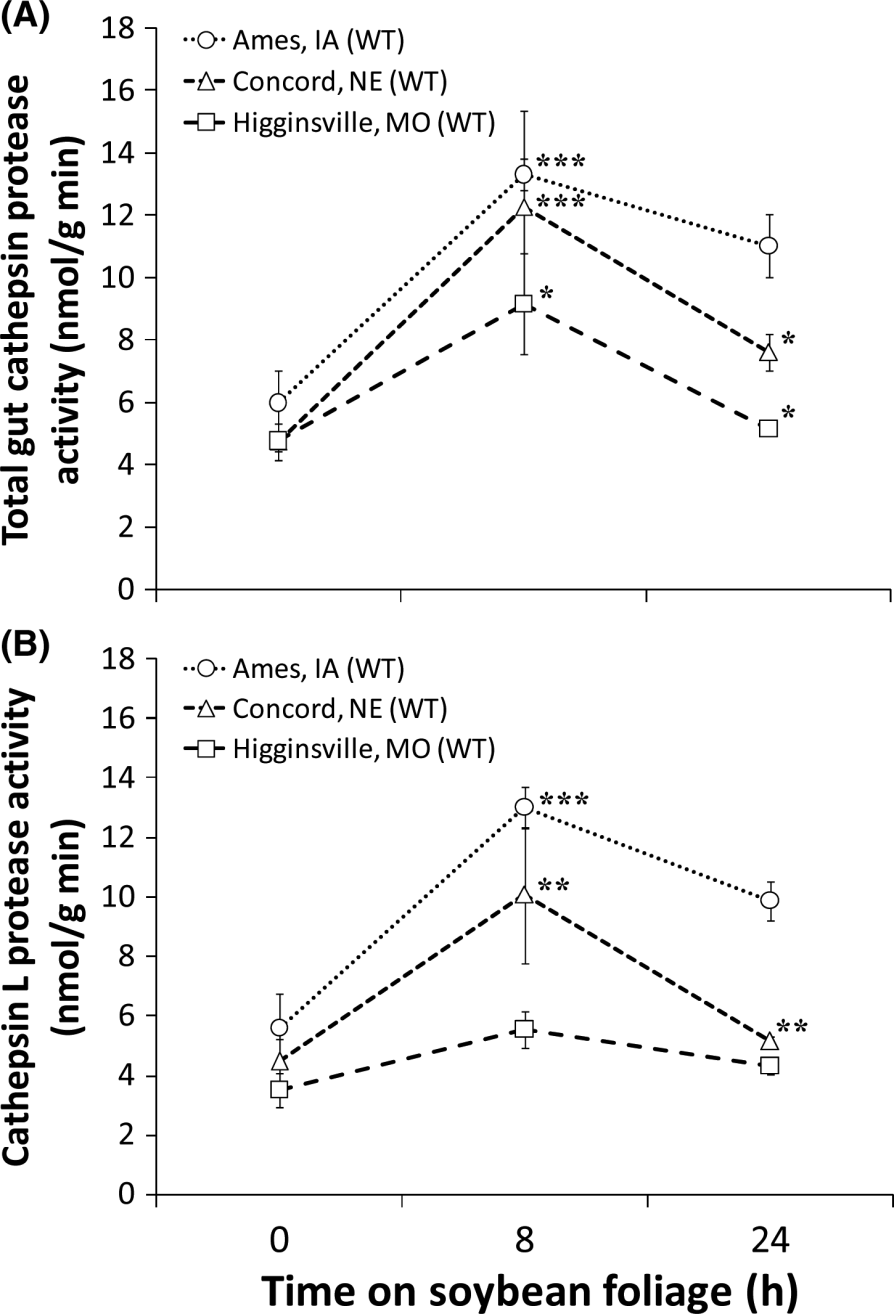
Cathepsin protease activity in guts of wild-type (WT) WCR adults that fed on soybeans in laboratory conditions. (A) Total cathepsin activity obtained using the chromogenic substrate p-Glu-Phe-Leu-pNA. (B) Cathepsin L-like activity obtained from the same extracts used in A (extracts were incubated with 1 μM of the cathepsin B specific inhibitor, CA-074). Samples were collected at 8 and 24 h of soybean herbivory. Time 0 baseline corresponds to beetles feeding on corn silks. Asterisks indicate significant difference of cathepsin activity compared with the previous time point (*P < 0.05; **P < 0.01; ***P < 0.001).

To determine whether altered cathepsin L protease activity can explain the differences in soybean damage and survivorship, excised guts from RR and WT WCR were analyzed. The field experiments showed that cathepsin L-like activity levels were higher in guts of beetles from RR (Urbana and LaSalle, IL) populations than in those from the WT (Iowa) population (*P* < 0.01; Fig. 4A). In the laboratory experiments, baseline cathepsin L-like protease activity in beetles that fed on corn-silk diet (0 h) was higher in RR (Minonk and Urbana, 20.4 and 14.0 nmol/g/min, respectively) than in WT populations (average of 5.0 nmol/g/min; *P* < 0.001; Fig. 4B). At 8 h of feeding on soybean foliage, total cathepsin activity was induced in beetles from WT populations (Fig. 5A). Although there was a peak in cathepsin L-like protease activity in the WT populations from Iowa and Nebraska (*P* < 0.01; Fig. 4B and Fig. 5B), cathepsin L-like protease activity in beetles from both RR populations at 8 h (Urbana, 15.6 nmol/g/min, and Minonk, 25.4 nmol/g/min) was significantly higher than that in beetles from the WT populations collected at Nebraska (10.1 nmol/g/min) and Missouri (5.6 nmol/g/min; *P* < 0.01; Fig. 4B). Beetles from the WT Iowa population had intermediate cathepsin L-like activity (13.0 nmol/g/min) that was significantly lower than the RR population from Minonk (*P* < 0.001), but was not different from the Urbana population (*P* > 0.05; Fig. 4B). After 8 h, cathepsin L activity declined rapidly until 24 h on soybean diet and then gradually until the end of the experiment (72 h) (Fig. 4B). The reduction of cathepsin L protease activity in WCR populations between 8 and 72 h on soybean diet is correlated with the increment in CystPI activity in soybean leaves, which occurs simultaneously (Fig 1). At 24 h of soybean herbivory, all activity curves showed a tendency to decrease to values similar to baseline levels measured on corn-silk diet (Fig. 4B), except in WT Iowa population where cathepsin L activity did not decrease to its baseline values and remained intermediate between baseline values of RR and Nebraska and Missouri WT populations. In addition, beyond 24 h and until the end of the experiment (72 h), cathepsin L activity remained relatively stable in all five populations.

Beetles from RR populations that fed on soybean foliage for 8 h had 25% higher cathepsin L-like gene (DvRS5) expression than those from WT populations (*P* < 0.05; Fig. 4 C). Among the five genes encoding WCR cathepsin L-like proteases that were analyzed in this study (Koiwa et al. 2000; Bown et al. 2004), only gene DvRS5 had high expression levels and differences between populations and treatments (considerably more PCR cycles were required to amplify the other four genes; Fig. S2A). Previous studies also have reported high expression levels of the cathepsin L gene DvRS5 in the WCR (Bown et al. 2004).

**Figure 6:**
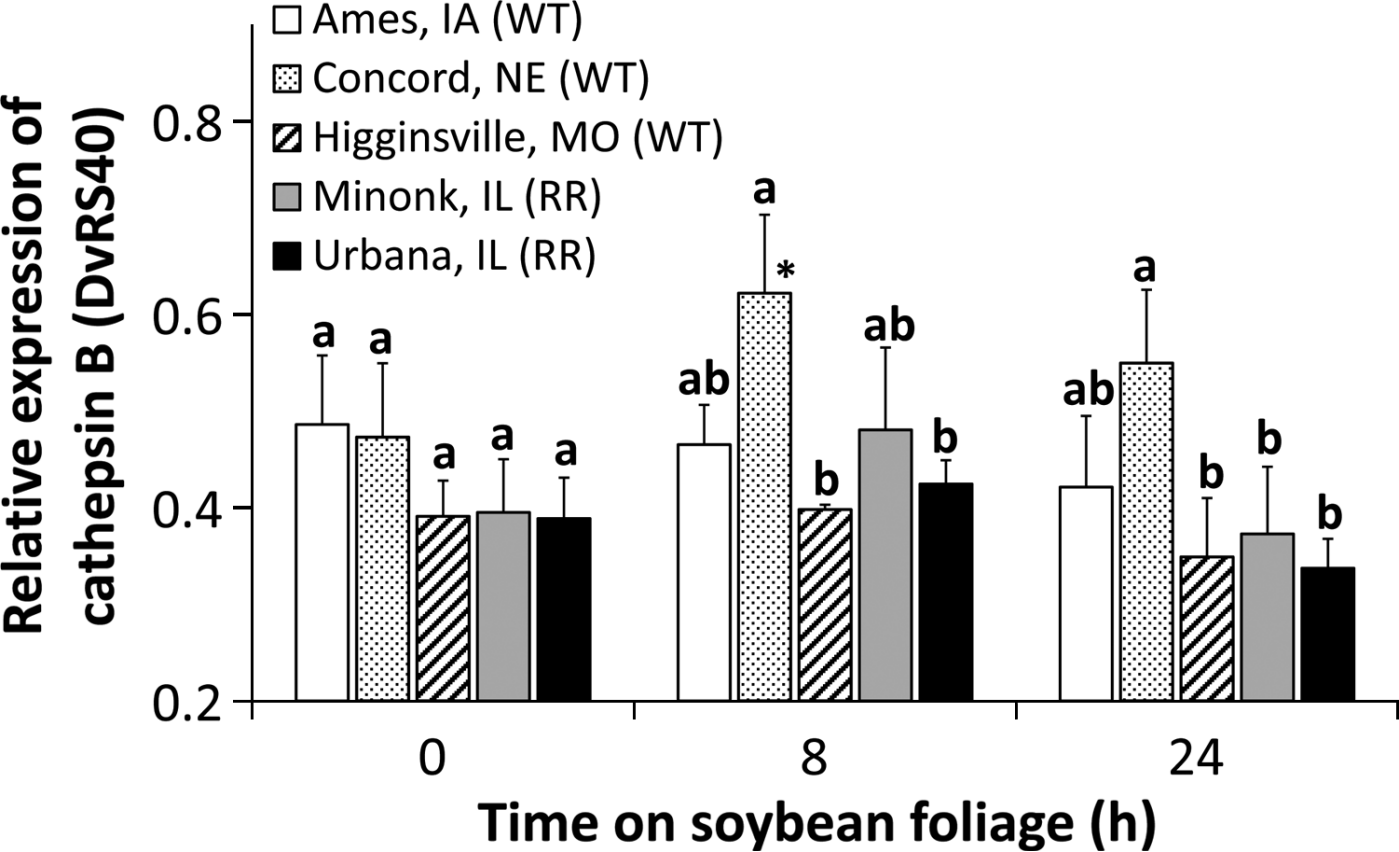
Quantitative RT-PCR expression analysis for cathepsin B-like (DvRS40) gene in guts of WCR adults. WCR adults fed for 0, 8, and 24 h on soybean foliage. Time points for each population represent three replicates each derived from five beetles. Values represent the band intensity generated by image analysis software for the results of quantitative RT-PCR relative to EF-1a (mean ± SE). Cathepsin B-like (DvRS40) gene accession number AJ583513. Bars bearing the same letter within each time point are not significantly different between populations at *P* < 0.05.

Expression of cathepsin B-like proteases may be an insect counter-defense against antinutritional factors, such as soybean CystPIs (Koo et al. 2008). Among soybean foliage-feeding WT beetles from NE, cathepsin B-like (DvRS40) gene expression was 32% higher for soybean-feeding versus corn silk-feeding adults (P < 0.05; Fig 5). WT (Nebraska) beetles that fed on soybean foliage for either 8 or 24 h had the highest cathepsin B-like protease expression and it was significantly greater than Higginsville and Urbana at 8 h and Higginsville, Urbana, and Minonk at 24 h of soybean herbivory (*P* < 0.05; Fig. 6). Although two cathepsin B-like genes were analyzed (DvRS40 and DvRS6) (Bown et al. 2004), expression of the DvRS6 gene was low and did not differ among populations and treatments (Fig. S2B).

**Figure 7:**
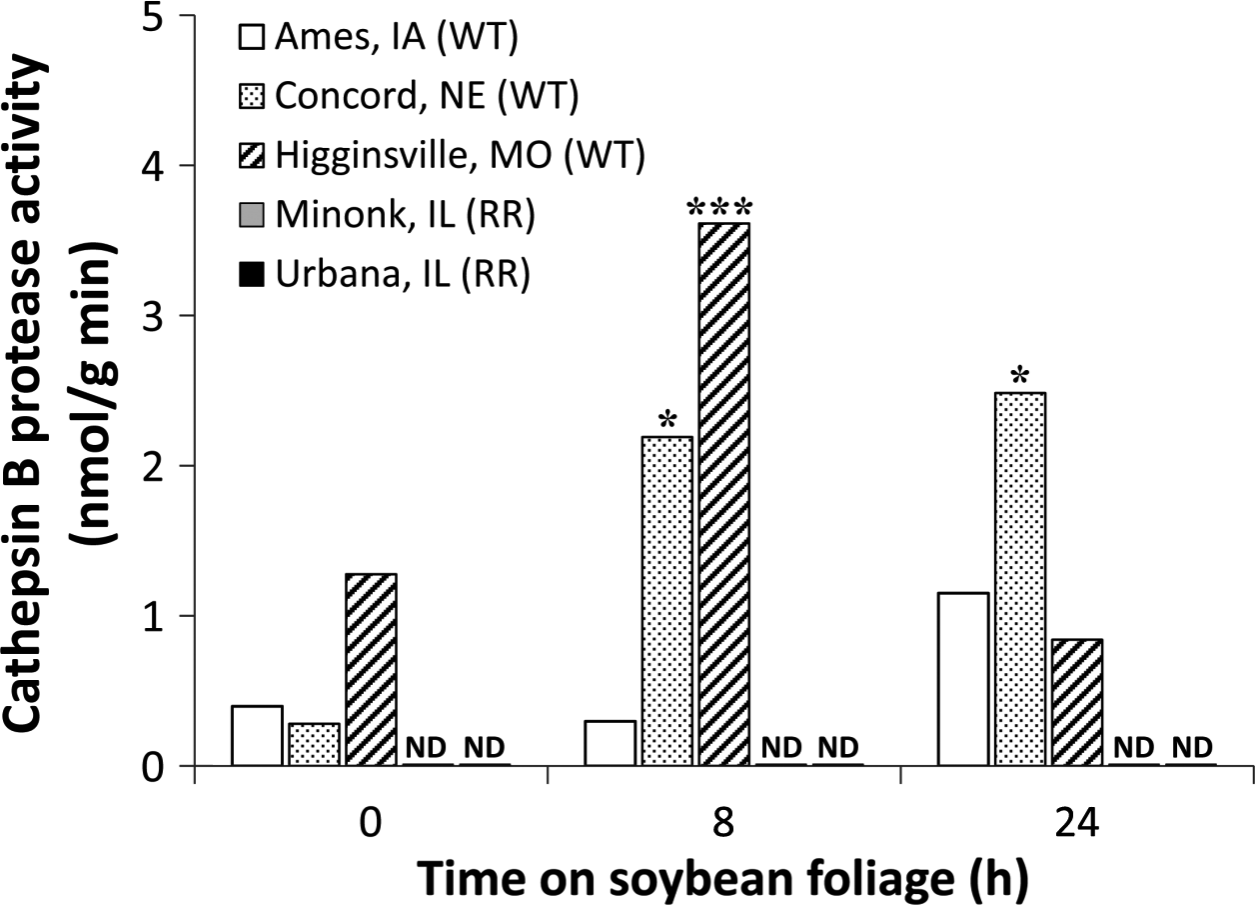
Estimated cathepsin B-like activity in guts of WCR that fed on soybean. Bars represent the portion of activity inhibited by the specific cathepsin B inhibitor, CA-074, using the chromogenic substrate p-Glu-Phe-Leu-pNA. WCR adults fed on soybean foliage for 8 and 24 h. Time 0 baseline corresponds to beetles feeding on corn silks. Cathepsin B-like activity was not detected (ND) in guts of WCR RR populations. Asterisks indicate populations/treatment combinations in which cathepsin B activity was significant (*P < 0.05; **P < 0.01; ***P < 0.001).

**Figure 8:**
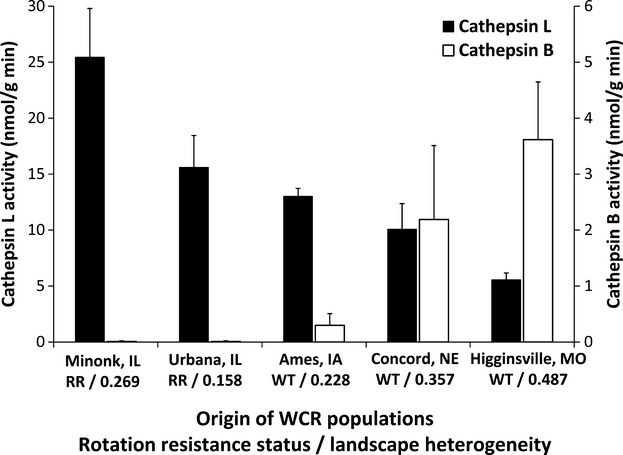
Patterns of cathepsin L- and B-like protease activities in RR and WT WCR populations. Filled bars represent cathepsin L-like activity after 8-hour soybean herbivory; empty bars represent cathepsin B-like activity after 8-h soybean herbivory. Populations were arranged by cathepsin L activity from high to low. WCR adults from locations where RR populations are present have the highest values of gut cathepsin L activity and no cathepsin B activity was detected, whereas in locations where WT populations are present, cathepsin L activity is lower and cathepsin B activity is significantly higher. In X axis are the geographical origin from which WCR populations were collected, their RR and WT status and the values of landscape heterogeneity defined as the proportion of land area planted with neither corn nor soybean.

Measurement of the difference in protease activity in the absence versus presence of the specific cathepsin B inhibitor, CA-074, can be used to estimate the fraction of activity attributable to cathepsin B-like proteases (Materials and Methods for details). Although experiments with substrate p-Glu-Phe-Leu-pNA underestimate cathepsin B activity, we detected differences in cathepsin B-like activity among WCR populations. Interestingly, cathepsin B activity was only ever induced in guts from WT beetles (populations from Nebraska and Missouri) that fed on soybean foliage for 8 and 24 h (*P* < 0.05; Fig. 7). No cathepsin B activity was detected in RR populations (Fig. 7). These results suggest that cathepsin B-like protease expression and activity only increased in beetles of WT populations as a response to high CystPI levels in soybean tissues. In addition, the patterns of cathepsin L-like and cathepsin B-like activities are consistent with the geographical areas where RR and WT are present (Fig. 8 and Fig. S1).

## Discussion

RR adults increased soybean intake and prolonged survival on a soybean foliage diet compared with WT populations. The results presented here demonstrate that the differences in behavior, physiology, protease activity, and gene expression in the gut of insects found in our study likely contribute to mechanisms that enable U.S. Corn Belt populations of RR WCR to circumvent soybean defenses and ultimately annual crop rotation. The link between this feeding behavior and gut physiology explains why there is a periodicity of RR WCR activity that includes a period of residence in soybeans. RR WCR females may spend several days in soybean fields before returning to cornfields (Mabry and Spencer 2003; Mabry et al. 2004). With the exception of a behavioral shift (Gray et al. 2009), for the last 15 years investigators have been unable to identify any differences between the WT and the RR WCR or mechanisms that allow this species to consume soybeans and survive longer in soybean fields (Miller et al. 2006, 2007; Knolhoff et al. 2010b). Here, we present eco-physiological, biochemical, and molecular evidence supporting higher tolerance of soybean herbivory in RR WCR due to an altered digestive proteolysis.

Despite significant soybean defenses against Coleopteran herbivory, RR WCR beetles that fed on soybeans had higher survivorship and consumed more leaf area than WT WCR beetles (Figs 1, 2, and 3). In accordance with our prediction, beetles from RR populations (regardless of whether they fed on soybean foliage) had higher cathepsin L-like protease expression and activity levels (threefold to fourfold greater) than beetles from WT populations (Fig. 4). As cathepsin L-like proteases are the main digestive enzymes in WCR and can be strongly inhibited by soybean CystPIs (Koiwa et al. 2000; Kim and Mullin 2003; Zavala et al. 2008), our results provide strong evidence that the elevated (relative to WT) constitutive cathepsin L-like protease activity in RR WCR allows them to survive and feed on soybean foliage for a few days, before their digestive enzymes are highly inhibited. Longer residence time of RR WCR in soybean increases their opportunity to feed on foliage and lay eggs in response to dietary stress (Branson and Krysan 1981; Mabry and Spencer 2003). In addition, consequences of dietary stress induced by digestive enzyme inhibition may be the trigger that initiates the eventual return to cornfields.

The fact that in field studies down-regulation of CystPI defenses in soybean plants increases digestive cysteine proteases in WCR beetles (Zavala et al. 2008) is consistent with the suggestion that WCR beetles responded to dietary CystPI by both qualitative and quantitative changes in their digestive proteases. Although activity of cathepsin L-like proteases induced in WT beetles that fed on soybean foliage did not reach that of RR beetles (Fig. 4B), WT beetles also induced expression and activity of cathepsin B-like (Figs 6 and Fig. 7). Cathepsin B plays a crucial role in the Coleopteran counter-defense against stress produced by CystPIs (Michaud et al. 1993; Cloutier et al. 2000; Koo et al. 2008). When challenged with diets containing soybean CystPI, cowpea bruchids induced activity and expression of cathepsin B-like proteases to compensate for inhibition of cathepsin L-like proteases (Koo et al. 2008). Interestingly, the enzymatic activity of cathepsin B-like proteases in bruchids is not inhibited by CystPI; the occluding loop in cathepsin B blocks the access of CystPI to its catalytic site cleft (Moon et al. 2004; Bayes et al. 2005). However, effective counter-defenses against dietary threats can be energetically costly, which slows insect growth and may decrease survivorship (Broadway 1996; Zavala et al. 2004; Chi et al. 2009). The metabolic cost of both cathepsins L- and B-like induction together with the inhibition activity of endoproteases and toxicity produced by CystPI in WT WCR beetles may explain their higher mortality and lower foliage consumption when fed on soybean foliage compared with RR WCR (Figs 2 and 3). On the basis of biotic system interactions, we do not rule out factors that may contribute to WCR performance such as other soybean defenses or the gut microbiome, but they are beyond the scope of this study.

The human-imposed changes in landscape heterogeneity associated with crop rotation created a vast patchwork of adjacent host and nonhost fields. The close integration of corn and soybean fields dramatically increased the probability that WCR would be exposed to the defenses of the nonhost soybean. Therefore, we hypothesize that higher levels of constitutive cathepsin L-like protease activity, along with reduced RR WCR female ovipositional fidelity to cornfields, were consequences of the selection imposed on WCR populations by broad-scale adoption of the corn/soybean crop rotation that decreased landscape heterogeneity (Table 1). However, we cannot be certain whether evolution of higher constitutive cathepsin L activity followed the reduction in ovipositional fidelity. The role of altered digestive proteolysis in adaptations to soybean herbivory is evident in the contrasting patterns of cathepsin L- and B-like protease activity between RR and WT WCR populations from different geographical origins (Fig. 8). While cathepsin L-like protease activity was high in RR WCR from near the Illinois epicenter of rotation resistance, the lowest cathepsin L-like activity was observed in the Missouri population. Compared with counties where the rotation-resistant populations were obtained (Fig. S1), the county where the wild-type Missouri population was collected has a more heterogeneous landscape with a much lower proportion of land area planted with corn and the greatest proportion of land area planted with neither corn nor soybean (Table 1). The relationships between landscape and WCR biotypes found in this study are in accordance with a mathematical model, which propose that greater landscape diversity can slow the spread of RR WCR (Onstad et al. 2003). The identification of a WCR population with intermediate constitutive cathepsin L activity levels supports the hypothesis that activity of digestive proteases in the guts of WCR might be selected by landscape heterogeneity. The Iowa WT WCR population, collected in a county (Story) with low landscape heterogeneity (Table 1) and separated from RR WCR expansion range by less than 100 km (Gray et al. 2009), had intermediate cathepsin L-like protease activity (Fig 8). In contrast, cathepsin B-like protease activity was only detected in WT WCR populations and was greatest in the Missouri population (Fig. 8). Taken together, these results may predict the imminent transition from a WT to a RR WCR phenotype in central Iowa.

**Table 1:** 2010 County level corn and soybean crop data for WCR collection sites

WCR collection site	WCR rotation resistance status	County land area (ha)	2010 Corn hectares planted^a^	2010 Soybean hectares planted^a^	Proportion of county land area in corn	Proportion of county land area that is neither corn nor soybean
Higginsville (Lafayette Co.), MO	WT	162,987	38,566	45,122	0.237	0.487
Concord (Dixon Co.), NE	WT	123,283	45,810	33,427	0.372	0.357
Ames (Story Co.), IA	WT	148,367	67,177	47,347	0.453	0.228
La Salle (La Salle Co.), IL	RR	293,937	135,972	85,792	0.463	0.246
Minonk (Woodford Co.), IL	RR	136,736	59,488	40,468	0.435	0.269
Urbana (Champaign Co.), IL	RR	258,168	118,166	99,146	0.458	0.158

a USDA-NASS (2010) Charts and Maps: Field Crops. Available: http://www.nass.usda.gov/Charts_and_Maps/Field_Crops/index.asp.

Our findings provide evidence of the possible cause and consequences of the contemporary evolution of the WCR in response to selection imposed by the modification of the agroecosystem. Examples of evolution occurring within decades or even a few generations are often associated with human-driven impacts including landscape modifications (Hendry and Kinnison 1999), such as the effects of rotated corn and soybean “monoculture” on behavior and digestive proteases activity of WCR populations in the U.S. Corn Belt. Moreover, our results make a novel connection between physiological and behavioral parameters associated with the transition of *Diabrotica virgifera* from a corn specialist to a biotype able to feed on both corn and soybeans. These new insights into the mechanism of WCR tolerance of soybean defenses transcend the issue of RR WCR diagnostics and management to link changes in gut proteolytic activity and insect behavior with landscape heterogeneity.

## Supporting Information

**Figure S1.** Collection sites of adult WCR populations. Areas where rotation-resistant behavioral phenotype has been reported are illustrated in gray on the map. Rotation-resistant populations (black circles) were collected in LaSalle (LaSalle County), Minonk (Woodford County), and Urbana (Champaign County), IL, whereas wild-type populations (open circles) were collected in Ames (Story County), IA, Concord (Dixon County), NE, and Higginsville (Lafayette County), MO.

**Figure S2.** Protease gene expression of *D. virgifera virgifera*. (A) Quantitative RT-PCR expression analysis of the five cathepsin L-like protease genes previously described in *D.v. virgifera* (Koiwa et al. 2000; Bown et al. 2004). (B) Quantitative RT-PCR expression analysis of the two cathepsin B-like protease genes of *D.v. virgifera* (Bown et al. 2004). Bracketed numbers indicate the number of PCR cycles required to obtain visible DNA bands within the exponential range of the amplification curve. The figures are a composite of gels for each clone and population and contain images spliced into place.

**Table S1.** Name and GenBank accession number of WCR cathepsin-like protease transcripts and sequence of both forward and reverse primers used in the experiments.

**Table S2.** Sequence of the WCR cathepsin-like cDNA fragments amplified for expression analysis.

**Table S3.** Pairwise comparisons of relative gene expression least squares means between WCR populations for each gene and treatment.
