# Supplementary figures and images for: Abnormally high digestive enzyme activity and gene expression explain the contemporary evolution of a Diabrotica biotype able to feed on soybeans
Source: Ecol Evol. 2012 Jul 19;2(8):2005–17. doi: 10.1002/ece3.331 (PMC3434003; doi:10.1002/ece3.331)

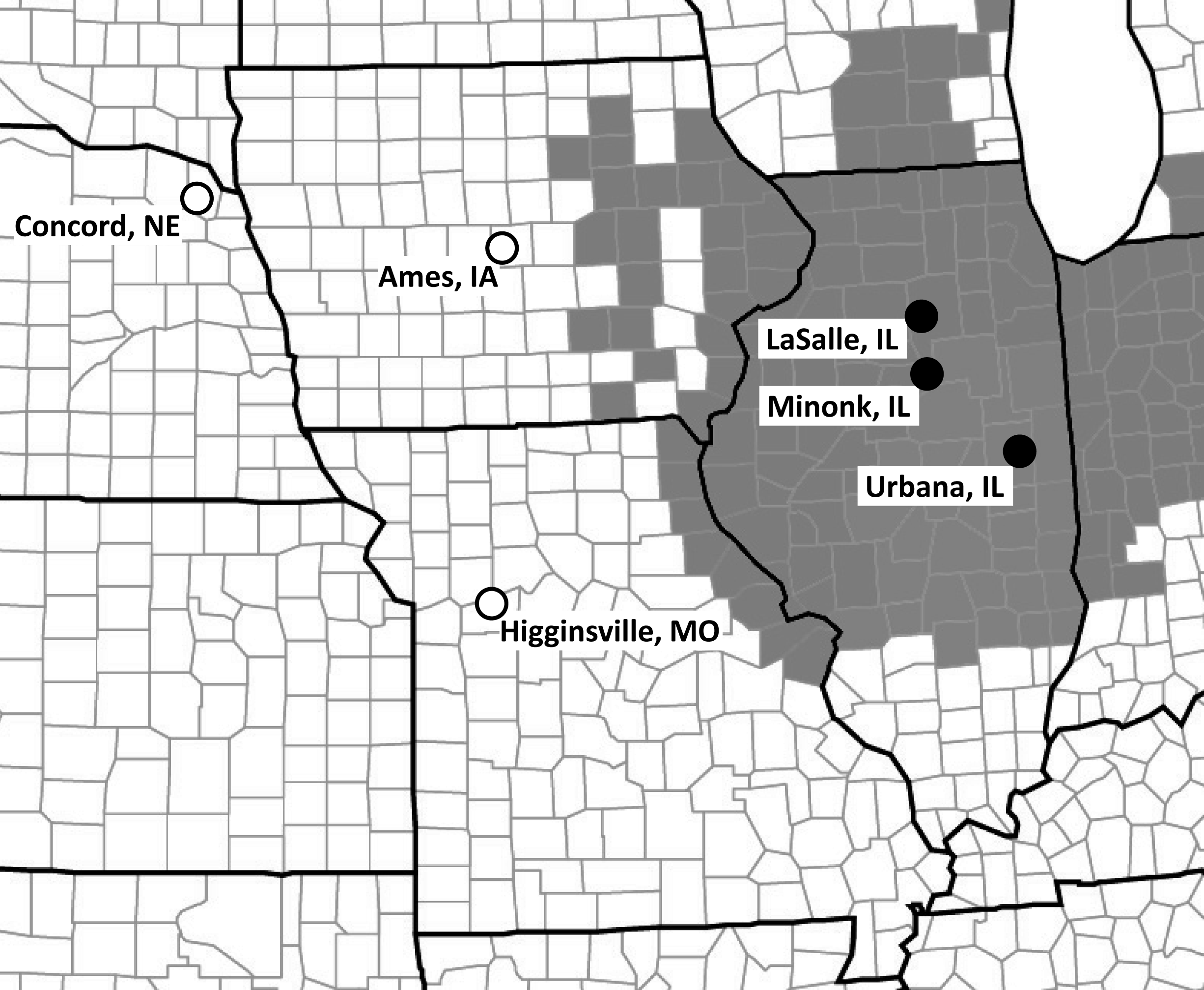

Supplement: Supplementary file 1 [file ece30002-2005-SD1.tif]

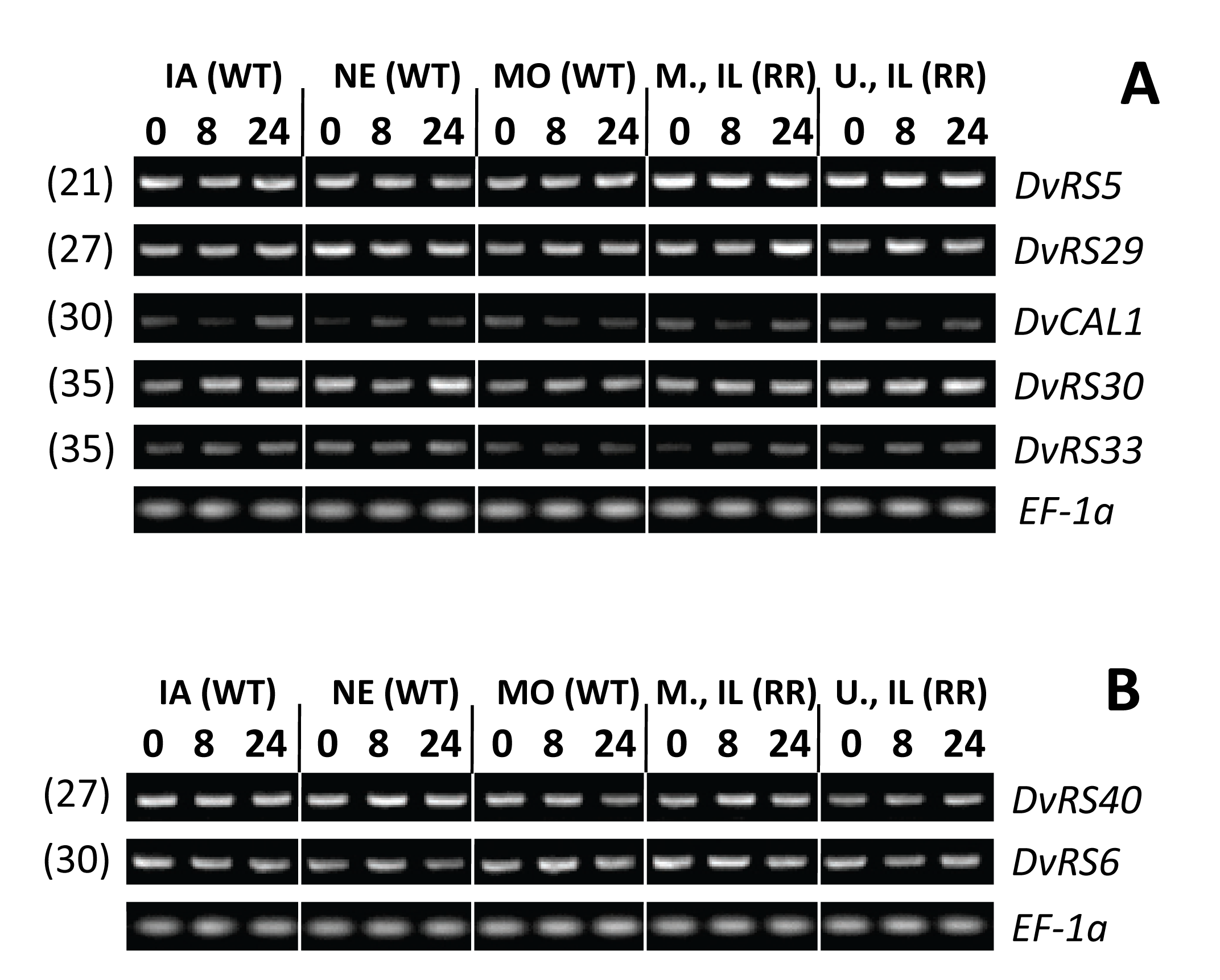

Supplement: Supplementary file 2 [file ece30002-2005-SD2.tif]
