# Supplementary material for: Abnormally high digestive enzyme activity and gene expression explain the contemporary evolution of a Diabrotica biotype able to feed on soybeans
Source: Ecol Evol. 2012 Jul 19;2(8):2005–17. doi: 10.1002/ece3.331 (PMC3434003; doi:10.1002/ece3.331)
Supplement: Supplementary file 3 [file ece30002-2005-SD3.doc]

**Supporting Information**

**Figures**

**
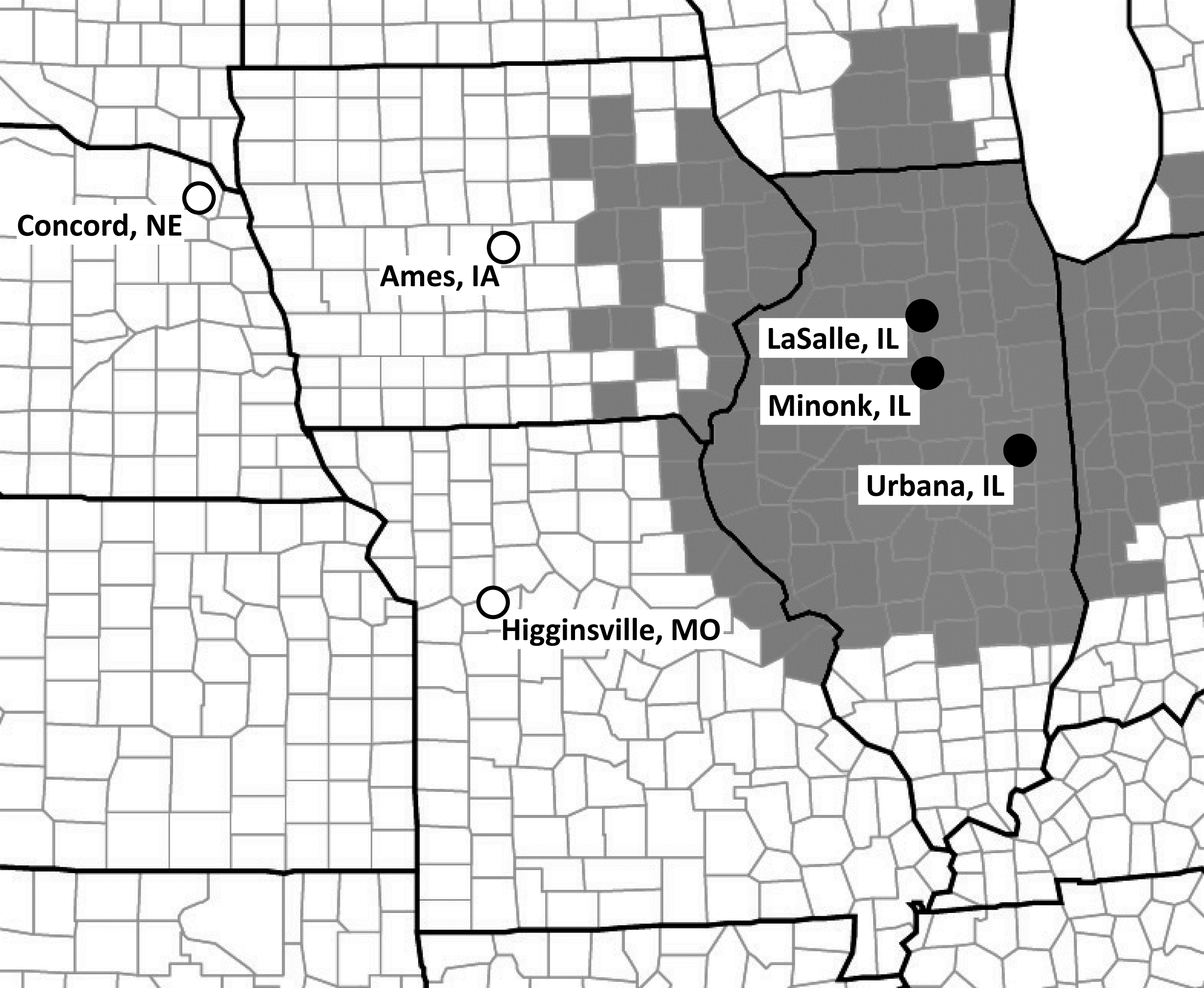
**

**Figure S1** Collection sites of adult WCR populations. Areas where rotation-resistant behavioral phenotype has been reported are illustrated in gray on the map. Rotation-resistant populations (black circles) were collected in LaSalle (LaSalle County), Minonk (Woodford County) and Urbana (Champaign County), IL, whereas wild-type populations (open circles) were collected in Ames (Story County), IA, Concord (Dixon County), NE and Higginsville (Lafayette County), MO.


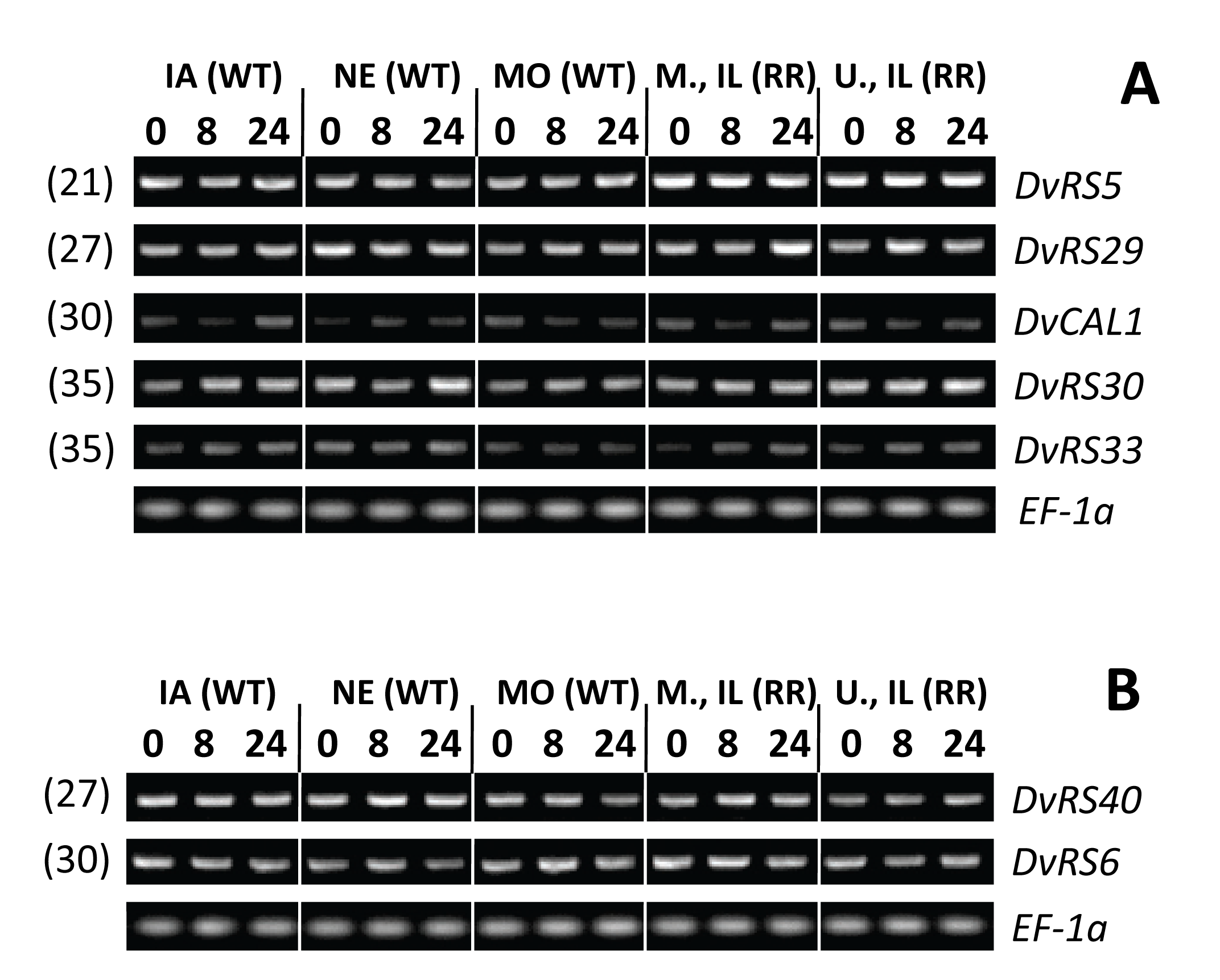


**Figure S2** Protease gene expression of *D. virgifera* *virgifera*. **(A)** Quantitative RT-PCR expression analysis of the five cathepsin L-like protease genes previously described in *D.v.* *virgifera* (Koiw*a et a*l. 2000; Bow*n et a*l. 2004). **(B)** Quantitative RT-PCR expression analysis of the two cathepsin B-like protease genes of *D.v. virgifera* (Bow*n et a*l. 2004). Bracketed numbers indicate the number of PCR cycles required to obtain visible DNA bands within the exponential range of the amplification curve. The figures are a composite of gels for each clone and population and contain images spliced into place.

**Tables**

| **Table S1** Name and GenBank accession number of WCR cathepsin-like protease transcripts and sequence of both forward and reverse primers used in the experiments. | | | |
| --- | --- | --- | --- |
| Name | Accession no. | Forward Primer | Reverse Primer |
| DvCAL1 | AF190653 | TTGCTGACATGACCCCAGA | AATCCAGTAGGGTGTGCCA |
| DVRS5 | AJ583508 | GCCAATCTAGGTGCCTTCGA | TGGAAACTGGACCTACGCT |
| DvRS6 | AJ583509 | ATCCACATGGACTGCAGGA | CGGATAGCATGACCTCCCA |
| DvRS29 | AJ583510 | AGTCCACCAACAATGGGCT | CACATACTGCAATTGGGCCA |
| DvRS30 | AJ583511 | AGTACGACTGGAACAGTGGA | GGGTATGTAGCGTCAGTGG |
| DvRS33 | AJ583512 | CTACACATTTGCTAGCCCCA | CCAGTTTACTCCCCAGGAG |
| DvRS40 | AJ583513 | ATGCATAGCATCCCAGGGA | TAGACTTGAGCAGGTGCAG |
| EF-1a |  | GGATGGCAAATCGAACGTAAA | GGGAAGGATAGCATCCAAAGC |

| **Table S2** Sequence of the WCR cathepsin-like cDNA fragments amplified for expression analysis. | | |
| --- | --- | --- |
| Name | Fragment length | Fragment sequence |
| DvCAL1 | 644 | TTGCTGACATGACCCCAGAAGAATTCAAAGCCAAGCTTGGTATGCAAGCTAAGAACATGCCCAAGATCAAGAAATCCCGTCATGTTAAAAATGTAAATGCTGAGGTTCCGGACTCCGTTGATTGGAGACAAAAAGGTGCCGTTTTAGGTGTTAAAGATCAAGGACAGTGTGGATCCTGCTGGGCTTTCAGTGCTACTGGTTCTCTTGAAGGACAAAACTACATCGTCAATGGAAAATCAGAACCCCTCAGTGAGCAGGAACTTCTGGATTGTTCCGTAGAATACGGAAATGGTGACTGCGATGAAGGTGGTCTTATGACCCTTGCTTTTGAGTTCGTCGAGGAAAACGGAATCGTATCTGAAGCTAGCTATCCGTACGAAGCTATCCAAGGAGATTGCAGAACAACCAATGACAAGGCAGTACTTCATATTCAAGGTTACAATGAAGTATATCCAAGTGAAGAAGCTTTGAGACAAGCTGTCGGTACTGTTGGTCCCATTTCTGCAGCTATTTGGGCTGAACCAATCCAGTTCTTCTCAAGCGGTATATATGACGACCCAAATTGCTTGAACTATGTCGAATACTTGGATCACGGAATTCTCGTCGTAGGTTACGGTGAAGAGAATGGCACACCCTACTGGATT |
| DVRS5 | 676 | GCCAATCTAGGTGCCTTCGAAAAATGGACCAGTTTTAAGGCAACCCATAACAAATCTTACAACGTTATTGAAGACAAACTTCGTTTCGCTGTTTTCCAAGACAACCTCAAAAAAATCGAGGAACACAATGCTAAATACGAAAGTGGAGAAGAAACCTACTACTTGGCTGTTAACAAATTCGCCGATTGGTCCAGCGCTGAATTCCAAGCTATGTTGGCCCGTCAGATGGCTAACAAGCCCAAACAATCCTTTATTGCAAAACACGTAGCCGATCCCAATGTCCAAGCTGTAGAAGAAGTTGATTGGAGAGATAGTGCCGTTTTGGGAGTCAAAGATCAAGGACAGTGTGGATCATGCTGGGCTTTCAGTACCACCGGATCCCTCGAAGGTCAACTCGCCATCCACAAAAATCAACGTGTTCCTCTCAGTGAACAAGAATTGGTAGACTGTGACACATCAAGAAATGCTGGTTGTAACGGAGGTTTGATGACAGATGCCTTTAACTATGTTAAACGCCATGGTCTCTCTTCCGAATCTCAATATGCATACACCGGCAGAGATGATCGCTGCAAGAATGTTGAGAACAAACCACTCTCTTCCATTAGTGGCTACGTAGAACTTGAAACAACTGAAGATGCACTCGCGTCCGCTGTTGCTAGCGTAGGTCCAGTTTCCA |
| DvRS6 | 738 | ATCCACATGGACTGCAGGAAGAAATTTTGCTCAGGACAAATCAATGGACTATATCATTAAATTGATGGGGGTATTACCTGACCATAAGAACTATATGCCTCCTGTATTGACACATAAACTAGAAGCTCTAGAAATTCCAGCCGATTTCGATGCTCGTCAGCAATGGCCACATTGCCCAACAATTCGGGAGATAAGAGATCAGGGATCATGCGGGTCATGTTGGGCTTTTGGAGCAGTGGAAGCTATGTCAGACAGAGTGTGCATTCATTCTAACGGTGAATCTAACTTCCATTTCTCATCAGATGATTTGGTTTCTTGCTGTTGGACATGTGGAATGGGATGTAATGGTGGATATCCTGGTGCAGCTTGGCATTATTGGGTCAGAAAAGGTCTTGTCTCTGGTGGTCAATATGGTACAAAACAGGGTTGCCGGCCATACGAAATTCCTCCTTGTGAACACCACACAAACGGATCACGTCCAGCCTGCGATGCTTCAGAAGGTAACACGCCAAAGTGTGCAAAATCATGCGAAAGTAACTACAAGATCAATTACAGTAATGACTTGCACTTTGGCTCTAAAGCATACTCCATTAGTAGTGACGTCAAACAAATACAGGCTGAAATATTACAAAATGGACCCGTAGAAGGAGCATTTAGCGTTTATGCTGATTTCGTGAACTATAAAACTGGGGTTTACCAACACATAAAAGGACAATTCTTGGGAGGTCATGCTATCCG |
| DvRS29 | 680 | AGTCCACCAACAATGGGCTCAATTTAAGGTAAACCATTCCAAGAAGTACGGACATCTTAAAGAAGAGCAAGTTCGCTTCCAAGTTTTCTCTCAAAATCTCCAGAAAATTGAACAGCACAATGCAAGATACCAGAATGGTGAAGTGTCCTTCTACTTGGGGGTTAATCAGTTCGCAGATATGACTTCAGAGGAATTCAAGGCTATGCTTGACTCCCAACTCATTCACAAGCCTAAGCGAGACATTACATCCCGCTTTGTAGCTGATCCTCAATTGACTGTTCCAGAATCAATTGACTGGAGAGAAAAGGGGGCAGTTAATCCCGTAAGGGACCAAGAGCAATGCGGATCATGTTGGGCATTTAGTGCAGCTGGTGCTCTTGAAGGACAAAGATTTTTAAAGGAGGGCAAACTAGAAGTACTGAGTACTCAACAGTTAGTAGATTGTTCCCGTGATTACAAAAATGAAGGCTGCAATGGTGGTTGGCCTCATTGGGCATATGACTACATCAAAGATAATGGCCTCTGTCTAGAGTCTAAGTACAAATATCAAGGATATGACGGTTACTACTGCAAAGAGTGTATTCCGGCTATCAAAAAAATCAATGGCTATTCATCTATAAATCAAACTGAAGAAGCACTTAAGGAGGCTGTGGGTACGGCTGGCCCAATTGCAGTATGTG |
| DvRS30 | 560 | AGTACGACTGGAACAGTGGAAGGAGCTTATTTTCTTAAAACAGGAAAATTGGTCTCTTTAAGTGAACAAAATCTCGTAGATTGCGCAAAAGAAGACTGTTATGGATGTTCAGGTGGGTACATGGACAAGGCATTAGAATACATTGAAACAGCTGGAGGAATAATGTCTGAAAACGATTATCCTTATGAAGGTATTGATGATAAATGCAGATTTGATAGCTCCAAAGTAGCTGCAAAAATTAGTAACTTTACTTATATTAAGAAGAACGATGAAGATGACCTGAAAAATGCCGTTATTGCAAAGGGACCCATATCAGTAGCTATTGATGCCAGTTTTAATTTTCAATTATATGATTCAGGAATACTCGATGATTCATCATGCTATAGTGACTTCAATTCTTTGAACCATGGAGTACTGGTTGTAGGCTATGGGACAGAGAAAGAGCAAGATTACTGGATCGTGAAAAACTCCTGGGGAGCTGACTGGGGAATGGATGGCTATATATGGATGAGCAGAAATAAAAATAACCAATGTGGTATTGCCACTGACGCTACATACCC |
| DvRS33 | 596 | CTACACATTTGCTAGCCCCACTAAGAGATCTACCATCAGCATTCGATTGGAGAGACAAGGGAGCTGTAACTGAGGTAAAAGATCAAGGAATGTGTGGCTCTTGTTGGACCTTTAGTACAACTGGGTCAGTAGAAGCAGCTCATTTTCTTAAAACTGGAAATCTGGTATCCTTAAGTGAACAAAATCTAGTAGATTGCGCAAAAGACACTTGCTATGGGTGTGGAGGTGGCTGGATGGACAAAGCTCTGGAATATATCGAAAAAGGAGGAATAATGTCTGAGAAGGATTATCCTTACGAAGGCGTAGATGACAACTGTAGATTTGATATTTCCAAAGTAGCTGCCAAGATCAGTAACTTTACTTACATTAAGAAAAATGATGAAGAAGATCTTAAAAACGCAGTTGCTGCAAAAGGTCCCATATCAGTAGCTATTGATGCTTCTGCTACATTCCAGTTATATGTATCAGGCATACTTGATGATACGGAATGCTCTAATGAATTTGACTCATTGAATCATGGTGTACTAGTTGTTGGATATGGCACAGAAAATGGAAAAGACTACTGGATCATCAAAAACTCCTGGGGAGTAAACTGG |
| DvRS40 | 633 | ATGCATAGCATCCCAGGGAAAGCTTAAAGTACCTGTTTCGGCTGAAAATCTATTGTCTTGCTGCGATTCCTGTGGATATGGATGCGAAGGAGGATATCCAACCATGGCATGGAGTTATTGGATAGATACAGGAATTACTACAGGAGGTCTTTATGGCAGCAAACAGGGTTGTCAACCTTATTCGCTTCAACCATGTGAACATCATACAGAGGGTAATAAAGTACAATGCAGCACTTTGGACTATGACACACCTTCTTGCAAACATAAATGTGACGATTCCGCACTCAATTACAAGTCCGAGTTAACTTTTGGTTCAGGTTCTGTGCGTAACTTCTATTCAGTTGCAAATATTCAAAAGGAAATATTGACGAATGGTCCGGTAGAAGCAGCTTTTGATGTGTACAGCGACTTCGTGAATTACAAAAGTGGTGTCTATCAACATGTTGCTGGAGAATATTTAGGAGGACATGCCGTCAGAATTTTAGGTTGGGGAGAAGAGAGTGGAGTCCCTTATTGGTTGGTTGCTAATTCATGGAATGAAGATTGGGGAGACAAGGGATTGTTTAAAATACGTCGCGGAAATAATGAATCAGGCTTCGAGGACTCTATTGTTGCTGCACCTGCTCAAGTCTA |

| **Table S3** Pairwise comparisons of relative gene expression least squares means between WCR populations for each gene and treatment. | | | | | | | |
| --- | --- | --- | --- | --- | --- | --- | --- |
| Gene^a^ | Treatment^b^ | Population 1 | Population 2 | Estimate | DF | t Value | Pr > |t| |
| DvCAL1 | All | Ames, IA | Concord, NE | -0.141 | 35 | -1.38 | 0.1770 |
| DvCAL1 | All | Ames, IA | Higginsville, MO | 0.103 | 35 | 0.79 | 0.4331 |
| DvCAL1 | All | Ames, IA | Minonk, IL | 0.117 | 35 | 1.14 | 0.2600 |
| DvCAL1 | All | Ames, IA | Urbana, IL | -0.051 | 35 | -0.49 | 0.6298 |
| DvCAL1 | All | Concord, NE | Higginsville, MO | 0.243 | 35 | 1.88 | 0.0691 |
| DvCAL1 | All | Concord, NE | Minonk, IL | 0.257 | 35 | 2.52 | 0.0163 |
| DvCAL1 | All | Concord, NE | Urbana, IL | 0.089 | 35 | 0.85 | 0.4000 |
| DvCAL1 | All | Higginsville, MO | Minonk, IL | 0.014 | 35 | 0.11 | 0.9158 |
| DvCAL1 | All | Higginsville, MO | Urbana, IL | -0.154 | 35 | -1.17 | 0.2480 |
| DvCAL1 | All | Minonk, IL | Urbana, IL | -0.168 | 35 | -1.60 | 0.1190 |
| DvCAL1 | 0 hs | Ames, IA | Concord, NE | -0.004 | 35 | -0.02 | 0.9843 |
| DvCAL1 | 0 hs | Ames, IA | Higginsville, MO | 0.170 | 35 | 0.77 | 0.4436 |
| DvCAL1 | 0 hs | Ames, IA | Minonk, IL | 0.092 | 35 | 0.52 | 0.6077 |
| DvCAL1 | 0 hs | Ames, IA | Urbana, IL | 0.172 | 35 | 0.98 | 0.3361 |
| DvCAL1 | 0 hs | Concord, NE | Higginsville, MO | 0.173 | 35 | 0.79 | 0.4343 |
| DvCAL1 | 0 hs | Concord, NE | Minonk, IL | 0.095 | 35 | 0.54 | 0.5940 |
| DvCAL1 | 0 hs | Concord, NE | Urbana, IL | 0.176 | 35 | 1.00 | 0.3265 |
| DvCAL1 | 0 hs | Higginsville, MO | Minonk, IL | -0.078 | 35 | -0.36 | 0.7229 |
| DvCAL1 | 0 hs | Higginsville, MO | Urbana, IL | 0.002 | 35 | 0.01 | 0.9913 |
| DvCAL1 | 0 hs | Minonk, IL | Urbana, IL | 0.081 | 35 | 0.46 | 0.6503 |
| DvCAL1 | 8 hs | Ames, IA | Concord, NE | -0.303 | 35 | -1.72 | 0.0951 |
| DvCAL1 | 8 hs | Ames, IA | Higginsville, MO | -0.032 | 35 | -0.14 | 0.8860 |
| DvCAL1 | 8 hs | Ames, IA | Minonk, IL | 0.032 | 35 | 0.18 | 0.8584 |
| DvCAL1 | 8 hs | Ames, IA | Urbana, IL | -0.363 | 35 | -2.05 | 0.0477 |
| DvCAL1 | 8 hs | Concord, NE | Higginsville, MO | 0.271 | 35 | 1.24 | 0.2239 |
| DvCAL1 | 8 hs | Concord, NE | Minonk, IL | 0.335 | 35 | 1.90 | 0.0663 |
| DvCAL1 | 8 hs | Concord, NE | Urbana, IL | -0.060 | 35 | -0.34 | 0.7382 |
| DvCAL1 | 8 hs | Higginsville, MO | Minonk, IL | 0.063 | 35 | 0.29 | 0.7740 |
| DvCAL1 | 8 hs | Higginsville, MO | Urbana, IL | -0.331 | 35 | -1.51 | 0.1401 |
| DvCAL1 | 8 hs | Minonk, IL | Urbana, IL | -0.394 | 35 | -2.23 | 0.0321 |
| DvCAL1 | 24 hs | Ames, IA | Concord, NE | -0.115 | 35 | -0.65 | 0.5192 |
| DvCAL1 | 24 hs | Ames, IA | Higginsville, MO | 0.171 | 35 | 0.78 | 0.4416 |
| DvCAL1 | 24 hs | Ames, IA | Minonk, IL | 0.227 | 35 | 1.29 | 0.2071 |
| DvCAL1 | 24 hs | Ames, IA | Urbana, IL | 0.037 | 35 | 0.19 | 0.8478 |
| DvCAL1 | 24 hs | Concord, NE | Higginsville, MO | 0.286 | 35 | 1.30 | 0.2011 |
| DvCAL1 | 24 hs | Concord, NE | Minonk, IL | 0.342 | 35 | 1.94 | 0.0609 |
| DvCAL1 | 24 hs | Concord, NE | Urbana, IL | 0.152 | 35 | 0.79 | 0.4334 |
| DvCAL1 | 24 hs | Higginsville, MO | Minonk, IL | 0.056 | 35 | 0.26 | 0.7984 |
| DvCAL1 | 24 hs | Higginsville, MO | Urbana, IL | -0.134 | 35 | -0.58 | 0.5650 |
| DvCAL1 | 24 hs | Minonk, IL | Urbana, IL | -0.190 | 35 | -0.99 | 0.3292 |
| DvRS5 | All | Ames, IA | Concord, NE | -0.041 | 22 | -0.72 | 0.4805 |
| DvRS5 | All | Ames, IA | Higginsville, MO | -0.035 | 22 | -0.56 | 0.5778 |
| DvRS5 | All | Ames, IA | Minonk, IL | -0.153 | 22 | -2.67 | 0.0139 |
| DvRS5 | All | Ames, IA | Urbana, IL | -0.183 | 22 | -3.19 | 0.0042 |
| DvRS5 | All | Concord, NE | Higginsville, MO | 0.006 | 22 | 0.11 | 0.9120 |
| DvRS5 | All | Concord, NE | Minonk, IL | -0.112 | 22 | -2.23 | 0.0362 |
| DvRS5 | All | Concord, NE | Urbana, IL | -0.142 | 22 | -2.83 | 0.0099 |
| DvRS5 | All | Higginsville, MO | Minonk, IL | -0.118 | 22 | -2.07 | 0.0508 |
| DvRS5 | All | Higginsville, MO | Urbana, IL | -0.148 | 22 | -2.59 | 0.0169 |
| DvRS5 | All | Minonk, IL | Urbana, IL | -0.030 | 22 | -0.59 | 0.5584 |
| DvRS5 | 0 hs | Ames, IA | Concord, NE | 0.031 | 22 | 0.32 | 0.7549 |
| DvRS5 | 0 hs | Ames, IA | Higginsville, MO | 0.082 | 22 | 0.77 | 0.4486 |
| DvRS5 | 0 hs | Ames, IA | Minonk, IL | -0.072 | 22 | -0.73 | 0.4703 |
| DvRS5 | 0 hs | Ames, IA | Urbana, IL | -0.060 | 22 | -0.61 | 0.5469 |
| DvRS5 | 0 hs | Concord, NE | Higginsville, MO | 0.051 | 22 | 0.52 | 0.6061 |
| DvRS5 | 0 hs | Concord, NE | Minonk, IL | -0.103 | 22 | -1.18 | 0.2494 |
| DvRS5 | 0 hs | Concord, NE | Urbana, IL | -0.091 | 22 | -1.04 | 0.3075 |
| DvRS5 | 0 hs | Higginsville, MO | Minonk, IL | -0.154 | 22 | -1.57 | 0.1298 |
| DvRS5 | 0 hs | Higginsville, MO | Urbana, IL | -0.142 | 22 | -1.45 | 0.1609 |
| DvRS5 | 0 hs | Minonk, IL | Urbana, IL | 0.012 | 22 | 0.14 | 0.8913 |
| DvRS5 | 8 hs | Ames, IA | Concord, NE | -0.074 | 22 | -0.76 | 0.4582 |
| DvRS5 | 8 hs | Ames, IA | Higginsville, MO | -0.075 | 22 | -0.71 | 0.4878 |
| DvRS5 | 8 hs | Ames, IA | Minonk, IL | -0.279 | 22 | -2.86 | 0.0092 |
| DvRS5 | 8 hs | Ames, IA | Urbana, IL | -0.278 | 22 | -2.85 | 0.0094 |
| DvRS5 | 8 hs | Concord, NE | Higginsville, MO | -0.001 | 22 | -0.01 | 0.9902 |
| DvRS5 | 8 hs | Concord, NE | Minonk, IL | -0.205 | 22 | -2.37 | 0.0272 |
| DvRS5 | 8 hs | Concord, NE | Urbana, IL | -0.204 | 22 | -2.35 | 0.0279 |
| DvRS5 | 8 hs | Higginsville, MO | Minonk, IL | -0.204 | 22 | -2.09 | 0.0485 |
| DvRS5 | 8 hs | Higginsville, MO | Urbana, IL | -0.203 | 22 | -2.08 | 0.0495 |
| DvRS5 | 8 hs | Minonk, IL | Urbana, IL | 0.001 | 22 | 0.01 | 0.9909 |
| DvRS5 | 24 hs | Ames, IA | Concord, NE | -0.080 | 22 | -0.82 | 0.4201 |
| DvRS5 | 24 hs | Ames, IA | Higginsville, MO | -0.111 | 22 | -1.04 | 0.3077 |
| DvRS5 | 24 hs | Ames, IA | Minonk, IL | -0.108 | 22 | -1.10 | 0.2827 |
| DvRS5 | 24 hs | Ames, IA | Urbana, IL | -0.210 | 22 | -2.15 | 0.0429 |
| DvRS5 | 24 hs | Concord, NE | Higginsville, MO | -0.031 | 22 | -0.31 | 0.7562 |
| DvRS5 | 24 hs | Concord, NE | Minonk, IL | -0.027 | 22 | -0.31 | 0.7558 |
| DvRS5 | 24 hs | Concord, NE | Urbana, IL | -0.130 | 22 | -1.49 | 0.1493 |
| DvRS5 | 24 hs | Higginsville, MO | Minonk, IL | 0.003 | 22 | 0.03 | 0.9727 |
| DvRS5 | 24 hs | Higginsville, MO | Urbana, IL | -0.099 | 22 | -1.01 | 0.3223 |
| DvRS5 | 24 hs | Minonk, IL | Urbana, IL | -0.102 | 22 | -1.18 | 0.2509 |
| DvRS6 | All | Ames, IA | Concord, NE | 0.050 | 36 | 0.76 | 0.4551 |
| DvRS6 | All | Ames, IA | Higginsville, MO | 0.047 | 36 | 0.55 | 0.5853 |
| DvRS6 | All | Ames, IA | Minonk, IL | 0.061 | 36 | 0.92 | 0.3661 |
| DvRS6 | All | Ames, IA | Urbana, IL | 0.046 | 36 | 0.68 | 0.4978 |
| DvRS6 | All | Concord, NE | Higginsville, MO | -0.004 | 36 | -0.04 | 0.9650 |
| DvRS6 | All | Concord, NE | Minonk, IL | 0.011 | 36 | 0.16 | 0.8736 |
| DvRS6 | All | Concord, NE | Urbana, IL | -0.005 | 36 | -0.07 | 0.9445 |
| DvRS6 | All | Higginsville, MO | Minonk, IL | 0.014 | 36 | 0.17 | 0.8657 |
| DvRS6 | All | Higginsville, MO | Urbana, IL | -0.001 | 36 | -0.01 | 0.9912 |
| DvRS6 | All | Minonk, IL | Urbana, IL | -0.015 | 36 | -0.23 | 0.8191 |
| DvRS6 | 0 hs | Ames, IA | Concord, NE | 0.154 | 36 | 1.34 | 0.1899 |
| DvRS6 | 0 hs | Ames, IA | Higginsville, MO | 0.110 | 36 | 0.77 | 0.4475 |
| DvRS6 | 0 hs | Ames, IA | Minonk, IL | 0.146 | 36 | 1.26 | 0.2142 |
| DvRS6 | 0 hs | Ames, IA | Urbana, IL | 0.056 | 36 | 0.48 | 0.6331 |
| DvRS6 | 0 hs | Concord, NE | Higginsville, MO | -0.044 | 36 | -0.31 | 0.7587 |
| DvRS6 | 0 hs | Concord, NE | Minonk, IL | -0.008 | 36 | -0.07 | 0.9433 |
| DvRS6 | 0 hs | Concord, NE | Urbana, IL | -0.099 | 36 | -0.85 | 0.3985 |
| DvRS6 | 0 hs | Higginsville, MO | Minonk, IL | 0.036 | 36 | 0.25 | 0.8027 |
| DvRS6 | 0 hs | Higginsville, MO | Urbana, IL | -0.054 | 36 | -0.38 | 0.7064 |
| DvRS6 | 0 hs | Minonk, IL | Urbana, IL | -0.090 | 36 | -0.78 | 0.4388 |
| DvRS6 | 8 hs | Ames, IA | Concord, NE | 0.041 | 36 | 0.36 | 0.7242 |
| DvRS6 | 8 hs | Ames, IA | Higginsville, MO | -0.036 | 36 | -0.25 | 0.8040 |
| DvRS6 | 8 hs | Ames, IA | Minonk, IL | -0.075 | 36 | -0.65 | 0.5194 |
| DvRS6 | 8 hs | Ames, IA | Urbana, IL | 0.063 | 36 | 0.55 | 0.5866 |
| DvRS6 | 8 hs | Concord, NE | Higginsville, MO | -0.077 | 36 | -0.54 | 0.5947 |
| DvRS6 | 8 hs | Concord, NE | Minonk, IL | -0.116 | 36 | -1.01 | 0.3210 |
| DvRS6 | 8 hs | Concord, NE | Urbana, IL | 0.022 | 36 | 0.19 | 0.8480 |
| DvRS6 | 8 hs | Higginsville, MO | Minonk, IL | -0.039 | 36 | -0.27 | 0.7851 |
| DvRS6 | 8 hs | Higginsville, MO | Urbana, IL | 0.099 | 36 | 0.69 | 0.4930 |
| DvRS6 | 8 hs | Minonk, IL | Urbana, IL | 0.138 | 36 | 1.20 | 0.2382 |
| DvRS6 | 24 hs | Ames, IA | Concord, NE | -0.044 | 36 | -0.38 | 0.7033 |
| DvRS6 | 24 hs | Ames, IA | Higginsville, MO | 0.066 | 36 | 0.46 | 0.6494 |
| DvRS6 | 24 hs | Ames, IA | Minonk, IL | 0.112 | 36 | 0.97 | 0.3377 |
| DvRS6 | 24 hs | Ames, IA | Urbana, IL | 0.018 | 36 | 0.16 | 0.8768 |
| DvRS6 | 24 hs | Concord, NE | Higginsville, MO | 0.110 | 36 | 0.77 | 0.4475 |
| DvRS6 | 24 hs | Concord, NE | Minonk, IL | 0.156 | 36 | 1.36 | 0.1837 |
| DvRS6 | 24 hs | Concord, NE | Urbana, IL | 0.062 | 36 | 0.54 | 0.5925 |
| DvRS6 | 24 hs | Higginsville, MO | Minonk, IL | 0.046 | 36 | 0.33 | 0.7469 |
| DvRS6 | 24 hs | Higginsville, MO | Urbana, IL | -0.048 | 36 | -0.33 | 0.7415 |
| DvRS6 | 24 hs | Minonk, IL | Urbana, IL | -0.094 | 36 | -0.82 | 0.4202 |
| DvRS29 | 0 hs | Ames, IA | Concord, NE | 0.130 | 36 | 1.00 | 0.3216 |
| DvRS29 | 0 hs | Ames, IA | Higginsville, MO | 0.036 | 36 | 0.23 | 0.8220 |
| DvRS29 | 0 hs | Ames, IA | Minonk, IL | 0.111 | 36 | 0.85 | 0.3985 |
| DvRS29 | 0 hs | Ames, IA | Urbana, IL | 0.085 | 36 | 0.66 | 0.5149 |
| DvRS29 | 0 hs | Concord, NE | Higginsville, MO | -0.094 | 36 | -0.58 | 0.5635 |
| DvRS29 | 0 hs | Concord, NE | Minonk, IL | -0.020 | 36 | -0.15 | 0.8812 |
| DvRS29 | 0 hs | Concord, NE | Urbana, IL | -0.045 | 36 | -0.35 | 0.7305 |
| DvRS29 | 0 hs | Higginsville, MO | Minonk, IL | 0.074 | 36 | 0.46 | 0.6470 |
| DvRS29 | 0 hs | Higginsville, MO | Urbana, IL | 0.049 | 36 | 0.30 | 0.7634 |
| DvRS29 | 0 hs | Minonk, IL | Urbana, IL | -0.026 | 36 | -0.20 | 0.8451 |
| DvRS29 | 8 hs | Ames, IA | Concord, NE | -0.168 | 36 | -1.30 | 0.2025 |
| DvRS29 | 8 hs | Ames, IA | Higginsville, MO | 0.103 | 36 | 0.64 | 0.5243 |
| DvRS29 | 8 hs | Ames, IA | Minonk, IL | -0.084 | 36 | -0.64 | 0.5235 |
| DvRS29 | 8 hs | Ames, IA | Urbana, IL | -0.116 | 36 | -0.89 | 0.3788 |
| DvRS29 | 8 hs | Concord, NE | Higginsville, MO | 0.272 | 36 | 1.69 | 0.0999 |
| DvRS29 | 8 hs | Concord, NE | Minonk, IL | 0.085 | 36 | 0.65 | 0.5173 |
| DvRS29 | 8 hs | Concord, NE | Urbana, IL | 0.053 | 36 | 0.41 | 0.6864 |
| DvRS29 | 8 hs | Higginsville, MO | Minonk, IL | -0.187 | 36 | -1.16 | 0.2528 |
| DvRS29 | 8 hs | Higginsville, MO | Urbana, IL | -0.219 | 36 | -1.36 | 0.1820 |
| DvRS29 | 8 hs | Minonk, IL | Urbana, IL | -0.032 | 36 | -0.25 | 0.8064 |
| DvRS29 | 24 hs | Ames, IA | Concord, NE | -0.069 | 36 | -0.53 | 0.5977 |
| DvRS29 | 24 hs | Ames, IA | Higginsville, MO | 0.036 | 36 | 0.22 | 0.8244 |
| DvRS29 | 24 hs | Ames, IA | Minonk, IL | -0.025 | 36 | -0.19 | 0.8511 |
| DvRS29 | 24 hs | Ames, IA | Urbana, IL | -0.155 | 36 | -1.19 | 0.2403 |
| DvRS29 | 24 hs | Concord, NE | Higginsville, MO | 0.105 | 36 | 0.65 | 0.5183 |
| DvRS29 | 24 hs | Concord, NE | Minonk, IL | 0.045 | 36 | 0.34 | 0.7333 |
| DvRS29 | 24 hs | Concord, NE | Urbana, IL | -0.086 | 36 | -0.66 | 0.5124 |
| DvRS29 | 24 hs | Higginsville, MO | Minonk, IL | -0.060 | 36 | -0.38 | 0.7093 |
| DvRS29 | 24 hs | Higginsville, MO | Urbana, IL | -0.191 | 36 | -1.19 | 0.2436 |
| DvRS29 | 24 hs | Minonk, IL | Urbana, IL | -0.130 | 36 | -1.00 | 0.3216 |
| DvRS29 | All | Ames, IA | Concord, NE | -0.036 | 36 | -0.48 | 0.6365 |
| DvRS29 | All | Ames, IA | Higginsville, MO | 0.059 | 36 | 0.62 | 0.5423 |
| DvRS29 | All | Ames, IA | Minonk, IL | 0.001 | 36 | 0.01 | 0.9903 |
| DvRS29 | All | Ames, IA | Urbana, IL | -0.062 | 36 | -0.82 | 0.4153 |
| DvRS29 | All | Concord, NE | Higginsville, MO | 0.094 | 36 | 0.99 | 0.3291 |
| DvRS29 | All | Concord, NE | Minonk, IL | 0.037 | 36 | 0.49 | 0.6279 |
| DvRS29 | All | Concord, NE | Urbana, IL | -0.026 | 36 | -0.35 | 0.7303 |
| DvRS29 | All | Higginsville, MO | Minonk, IL | -0.058 | 36 | -0.61 | 0.5486 |
| DvRS29 | All | Higginsville, MO | Urbana, IL | -0.120 | 36 | -1.26 | 0.2150 |
| DvRS29 | All | Minonk, IL | Urbana, IL | -0.063 | 36 | -0.84 | 0.4085 |
| DvRS40 | All | Ames, IA | Concord, NE | -0.052 | 33 | -1.25 | 0.2186 |
| DvRS40 | All | Ames, IA | Higginsville, MO | 0.154 | 33 | 3.20 | 0.0030 |
| DvRS40 | All | Ames, IA | Minonk, IL | 0.041 | 33 | 1.10 | 0.2791 |
| DvRS40 | All | Ames, IA | Urbana, IL | 0.074 | 33 | 1.97 | 0.0577 |
| DvRS40 | All | Concord, NE | Higginsville, MO | 0.206 | 33 | 4.14 | 0.0002 |
| DvRS40 | All | Concord, NE | Minonk, IL | 0.094 | 33 | 2.25 | 0.0311 |
| DvRS40 | All | Concord, NE | Urbana, IL | 0.126 | 33 | 3.04 | 0.0047 |
| DvRS40 | All | Higginsville, MO | Minonk, IL | -0.112 | 33 | -2.34 | 0.0255 |
| DvRS40 | All | Higginsville, MO | Urbana, IL | -0.080 | 33 | -1.66 | 0.1062 |
| DvRS40 | All | Minonk, IL | Urbana, IL | 0.033 | 33 | 0.87 | 0.3929 |
| DvRS40 | 0 hs | Ames, IA | Concord, NE | 0.052 | 33 | 0.73 | 0.4716 |
| DvRS40 | 0 hs | Ames, IA | Higginsville, MO | 0.171 | 33 | 2.11 | 0.0428 |
| DvRS40 | 0 hs | Ames, IA | Minonk, IL | 0.091 | 33 | 1.40 | 0.1721 |
| DvRS40 | 0 hs | Ames, IA | Urbana, IL | 0.097 | 33 | 1.49 | 0.1452 |
| DvRS40 | 0 hs | Concord, NE | Higginsville, MO | 0.119 | 33 | 1.40 | 0.1701 |
| DvRS40 | 0 hs | Concord, NE | Minonk, IL | 0.039 | 33 | 0.55 | 0.5827 |
| DvRS40 | 0 hs | Concord, NE | Urbana, IL | 0.046 | 33 | 0.64 | 0.5246 |
| DvRS40 | 0 hs | Higginsville, MO | Minonk, IL | -0.080 | 33 | -0.98 | 0.3327 |
| DvRS40 | 0 hs | Higginsville, MO | Urbana, IL | -0.073 | 33 | -0.91 | 0.3716 |
| DvRS40 | 0 hs | Minonk, IL | Urbana, IL | 0.006 | 33 | 0.10 | 0.9242 |
| DvRS40 | 8 hs | Ames, IA | Concord, NE | -0.118 | 33 | -1.67 | 0.1053 |
| DvRS40 | 8 hs | Ames, IA | Higginsville, MO | 0.143 | 33 | 1.76 | 0.0870 |
| DvRS40 | 8 hs | Ames, IA | Minonk, IL | -0.015 | 33 | -0.23 | 0.8165 |
| DvRS40 | 8 hs | Ames, IA | Urbana, IL | 0.041 | 33 | 0.63 | 0.5362 |
| DvRS40 | 8 hs | Concord, NE | Higginsville, MO | 0.261 | 33 | 3.08 | 0.0042 |
| DvRS40 | 8 hs | Concord, NE | Minonk, IL | 0.103 | 33 | 1.45 | 0.1564 |
| DvRS40 | 8 hs | Concord, NE | Urbana, IL | 0.159 | 33 | 2.24 | 0.0319 |
| DvRS40 | 8 hs | Higginsville, MO | Minonk, IL | -0.158 | 33 | -1.95 | 0.0594 |
| DvRS40 | 8 hs | Higginsville, MO | Urbana, IL | -0.102 | 33 | -1.26 | 0.2162 |
| DvRS40 | 8 hs | Minonk, IL | Urbana, IL | 0.056 | 33 | 0.86 | 0.3965 |
| DvRS40 | 24 hs | Ames, IA | Concord, NE | -0.090 | 33 | -1.27 | 0.2144 |
| DvRS40 | 24 hs | Ames, IA | Higginsville, MO | 0.148 | 33 | 1.83 | 0.0769 |
| DvRS40 | 24 hs | Ames, IA | Minonk, IL | 0.049 | 33 | 0.74 | 0.4621 |
| DvRS40 | 24 hs | Ames, IA | Urbana, IL | 0.084 | 33 | 1.29 | 0.2065 |
| DvRS40 | 24 hs | Concord, NE | Higginsville, MO | 0.238 | 33 | 2.80 | 0.0084 |
| DvRS40 | 24 hs | Concord, NE | Minonk, IL | 0.138 | 33 | 1.95 | 0.0597 |
| DvRS40 | 24 hs | Concord, NE | Urbana, IL | 0.174 | 33 | 2.45 | 0.0197 |
| DvRS40 | 24 hs | Higginsville, MO | Minonk, IL | -0.099 | 33 | -1.23 | 0.2285 |
| DvRS40 | 24 hs | Higginsville, MO | Urbana, IL | -0.064 | 33 | -0.79 | 0.4360 |
| DvRS40 | 24 hs | Minonk, IL | Urbana, IL | 0.036 | 33 | 0.54 | 0.5897 |
| ^a^ No statistical analysis was conducted for DvRS30 and DvRS33. | | | | | | | |
| ^b^ Pairwise comparisons between the main effect of WCR populations (All) and for each treatment (0 h,8 h and 24 h). | | | | | | | |
